# The morphogenesis-related NDR kinase pathway of *Colletotrichum orbiculare* is required for translating plant surface signals into infection-related morphogenesis and pathogenesis

**DOI:** 10.1371/journal.ppat.1006189

**Published:** 2017-02-01

**Authors:** Sayo Kodama, Junya Ishizuka, Ito Miyashita, Takaaki Ishii, Takumi Nishiuchi, Hideto Miyoshi, Yasuyuki Kubo

**Affiliations:** 1 Graduate School of Life and Environmental Sciences, Kyoto Prefectural University, Kyoto, Japan; 2 Division of Functional Genomics, Advanced Science Research Center, Kanazawa University, Kanazawa, Japan; 3 Graduate School of Agriculture, Kyoto University, Kyoto, Japan; University of Nebraska-Lincoln, UNITED STATES

## Abstract

Plant infection by pathogenic fungi involves the differentiation of appressoria, specialized infection structures, initiated by fungal sensing and responding to plant surface signals. How plant fungal pathogens control infection-related morphogenesis in response to plant-derived signals has been unclear. Here we showed that the morphogenesis-related NDR kinase pathway (MOR) of the cucumber anthracnose fungus *Colletotrichum orbiculare* is crucial for appressorium development following perception of plant-derived signals. By screening of random insertional mutants, we identified that the MOR element CoPag1 (Perish-in-the-absence-of-*GYP1*) is a key component of the plant-derived signaling pathway involved in appressorium morphogenesis. Constitutive activation of the NDR kinase CoCbk1 (Cell-wall-biosynthesis-kinase-1) complemented *copag1* defects. Furthermore, *copag1* deletion impaired CoCbk1 phosphorylation, suggesting that CoPag1 functions *via* CoCbk1 activation. Searching for the plant signals that contribute to appressorium induction *via* MOR, we found that the cutin monomer *n*-octadecanal, degraded from the host cuticle by conidial esterases, functions as a signal molecule for appressorium development. Genome-wide transcriptional profiling during appressorium development revealed that MOR is responsible for the expression of a subset of the plant-signal-induced genes with potential roles in pathogenicity. Thus, MOR of *C*. *orbiculare* has crucial roles in regulating appressorium development and pathogenesis by communicating with plant-derived signals.

## Author summary

Phytopathogenic fungi cause many of the most serious crop diseases. Many fungal pathogens form specialized infection structures, called appressoria in response to plant surface signals. Networks of protein kinase-based signaling pathways regulate a wide variety of key morphological processes including appressorium development in several fungal pathogens. However, the precise link between plant signals and fungal intracellular transduction is poorly understood. Here, we report on the identification of a native molecule and a cognate signal transduction pathway involved in appressorium morphogenesis of the cucumber anthracnose fungus *Colletotrichum orbiculare*. We demonstrate that the widely conserved morphogenesis-related NDR kinase pathway (MOR) of *C*. *orbiculare* regulates infection structure development triggered by plant-derived signals and involves in pathogenesis. The cutin monomer *n*-octadecanal released from the host cuticle by conidial esterases functions as the signal molecule for appressorium development *via* MOR. Inactivating MOR resulted in downregulation of the plant-signal-induced genes including fungal secreted proteins that potentially facilitate infection. Thus, MOR is the crucial coordinator connecting plant surface signals with infection-related morphogenesis and pathogenesis. While previous reports have revealed that MOR is crucial for controlling cell polarity and differentiation in other fungi, our study provides its new role in the interaction of fungal pathogens with host plant.

## Introduction

*Colletotrichum orbiculare* (syn. *C*. *lagenarium*) is a plant pathogenic fungus causing anthracnose disease in cucumber (*Cucumis sativus*). Like other *Colletotrichum* species, *C*. *orbiculare* infects host plants hemibiotrophically: first, *C*. *orbiculare* forms melanized appressoria that mediate the direct penetration of host epidermal cells using a combination of mechanical force and enzymatic degradation, then it develops biotrophic hyphae inside living epidermal cells, and finally forms necrotrophic hyphae that kill and destroy host tissues [1, 2]. In many plant pathogenic fungi including *Colletotrichum* species, adhesion to the plant surface is the first step to initiate the infection process [3]. An extracellular matrix that surrounds spores contributes to their attachment and creates a host surface environment for successful penetration. The matrix of the rust fungus *Uromyces viciae-fabae* and the powdery mildew fungus *Blumeria graminis*, also contains esterases and assists in initiating the infection process as well as in adhesion to the host cuticle [3]. After adhesion of the spores, appressorium differentiation is triggered by fungal perception of various physical and chemical signals from the host surface [3, 4]. Previous studies have revealed that physical signals including hardness and hydrophobicity stimulate conidium germination and appressorium formation. Besides such physical stimuli, chemical signals such as leaf waxes and cutin monomers also induce appressorium formation in several plant pathogenic fungi. Analysis of the genome-wide expression profiles of *C*. *higginsianum* revealed that appressoria formed *in vitro* are morphologically indistinguishable from those *in planta*, their transcriptomes are substantially different. This indicates that these specialized cells are highly responsive to plant-derived signals that are perceived before penetration [2].

During infection by *C*. *orbiculare*, cell polarity is established and maintained in each developmental stage: conidial germ tube emergence, germ tube elongation, penetration peg formation from the appressorium, and hyphal tip extension inside the host plant [1, 5]. Thus, establishment of cell polarity is essential for morphogenesis of infection structures and pathogenicity. In our previous study, we elucidated that *CoKEL2*, a homolog of *Schizosaccharomyces pombe tea1*, a landmark protein of microtubule plus ends [6], localized at the apex of vegetative hyphae and germ tubes [7]. CoKel2 is required for proper morphogenesis of appressoria on artificial surfaces, but is dispensable on the host plant surface, suggesting a bypass pathway was activated by plant-derived signals independent of CoKel2 function [7]. Regulatory processes necessary for appressorium formation such as the cAMP/PKA and MAP kinase signaling cascades responding to plant-surface signals including physical and chemical signals have been described for *C*. *orbiculare* [1], however, precise dissection about the signal cascades that discriminate perception of those signals has largely been obscure in fungal plant pathogens.

Networks of protein kinase-based signaling pathways regulate a wide variety of key morphological processes. Members of the conserved NDR (nuclear Dbf2-related) kinases are important for controlling cell polarity and differentiation in various organisms [8]. Previously, studies in fungi and higher eukaryotes on NDR kinases have discovered the morphogenesis-related NDR kinase network (MOR) [9], which is also called RAM (regulation of Ace2 and morphogenesis). The central component of the system is an NDR kinase, Cbk1 in *Saccharomyces cerevisiae*, Orb6 in *S pombe*, and COT1 in *Neurospora crassa*, that associates with a regulatory subunit Mob2 [10–13]. This complex is activated by a Kic1/Nak1-related germinal center (GC) kinase and coordinated by Hym1/Pmo25-related and Tao3 (also known as Pag1)/Mor2/FRY-related scaffolding proteins [9, 14].

Although the MOR elements except Ace2 are highly conserved among eukaryotes, a deficiency in their functions can result in diverse cellular responses. While mutants in ascomycete yeasts *S*. *cerevisiae* [10] and *S*. *pombe* [11] display defects in cell polarity, mutations in the basidiomycetes *Cryptococcus neoformans* [15] and *Ustilago maydis* [16] result in hyperpolarized growth. In the filamentous ascomycetes including *N*. *crassa* [17] and *Aspergillus nidulans* [18], mutants are blocked in hyphal tip extension and display hyperbranched growth. Thus, despite the MOR components being conserved among various fungi, the inputs and outputs to and from the MOR central core are most likely species-specific. Although the possible link between NDR and MAP kinases pathway was reported in *N*. *crassa* [19], the upstream activators and downstream targets of MOR have been poorly studied [9], and further comparative analyses of cellular signaling context are still required.

Here, we demonstrate that the MOR of *C*. *orbiculare* plays a crucial role in the signal transduction pathway for appressorium development that is specifically induced by plant-derived cues. We also show that the signal molecule for appressorium induction *via* MOR is the cutin monomer *n*-octadecanal degraded from the host plant cuticle by conidial surface esterases. Furthermore, based on genome-wide gene expression analysis, we reveal that the MOR of *C*. *orbiculare* contributes to the regulation of a subset of the plant-signal-induced genes with potential roles in pathogenicity.

## Results

### Identification of the novel gene *C*. *orbiculare PAG1*, a functional ortholog of *S*. *cerevisiae* RAM component *TAO3*

To determine the specific components of the plant-derived signaling pathway for appressorium differentiation, we screened 10,021 insertional mutants in the *cokel2Δ* background and obtained 38 mutants that formed abnormal appressoria on the host plant and were reduced in pathogenicity compared with *cokel2Δ*. We next examined whether the reintroduction of *CoKEL2* into those mutants restored normal appressorium formation on artificial substrates to isolate mutants that have defects in the *CoKEL2*-independent and the plant-derived signaling pathways for appressorium formation. Eventually, six insertional mutants named as pathogenesis deficient mutants (PDM) potentially defective in the plant-derived signaling pathway were obtained (S1 Fig). Microscopic analysis showed that the PDMs formed abnormal appressoria with lateral germination both on artificial surface and host plant surface (S1A–S1D Fig). Consequently, PDMs were reduced in pathogenicity compared with *cokel2Δ* (S1E Fig). The candidate mutated gene was determined by whole genome sequencing of those mutants. Of the identified candidates, the mutated gene of PDM-4 showed high homology to *TAO3* (Transcriptional-Activator-of-*O**CH1*), also known as *PAG1* (Perish-in-the-Absence-of-*G**YP1*) in *Saccharomyces cerevisiae*, and we named this gene *CoPAG1*. In *S*. *cerevisiae*, Tao3 is one of the components of RAM, a signaling cascade that is involved in the maintenance of cell polarity, separation and cellular morphogenesis [9, 14, 20].

*CoPAG1* putatively encodes a 2419-amino-acid protein with MOR2-PAG1 domains, which are conserved in Pag1 homologous proteins (S2A Fig). CoPag1 homologs are conserved among other fungi and eukaryotes [9, 20]. All of its homologs are large proteins with more than 2000 amino acids and MOR2-PAG1 domains. In a phylogenetic tree generated on the basis of the derived amino acid sequences showed that the proteins of filamentous ascomycetes form a single clade, different from yeasts, basidiomcetes and other eukaryotes (S2B Fig). Furthermore, *CoPAG1* was confirmed as an ortholog of *S*. *cerevisiae TAO3* by complementation of *S*. *cerevisiae* strain FLY1004, carrying a deletion of *TAO3* (S2C and S2D Fig). Deletion of any of the RAM component genes including *TAO3* leads to two distinct phenotypes: (1) the accumulation of large aggregates of cells that are unable to degrade the septum between the mother and daughter cells, (2) a loss of polarity [10]. In the *tao3Δ* mutant, cells are aggregated and round (mean axial ratio 1.03). In contrast, the yeast transformants expressing the *CoPAG1* cDNA formed no aggregates and had a mean axial ratio 1.17 comparable with those of the wild type strain W303-1A (mean axial ratio 1.18) and the *tao3Δ* strain transformed with *TAO3* (mean axial ratio 1.17). Thus, the yeast complementation experiments showed that *PAG1* of *C*. *orbiculare* is a functional ortholog of *S*. *cerevisiae TAO3*.

### CoPag1 is a key component of the plant-derived signaling pathway involved in appressorium morphogenesis

**Fig 1:**
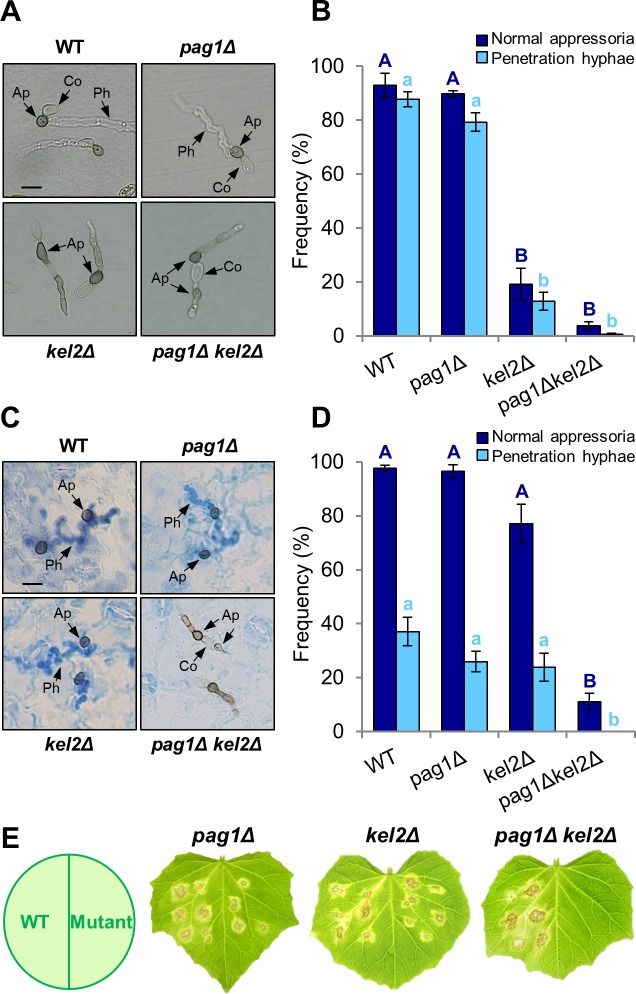
CoPag1 is a key component of the signal transduction pathway for appressorium formation induced by plant-derived cues. (A) Development of infection structures of wild type strain 104-T (WT) and *cokel2* and *copag1* mutant strains on cellulose membranes at 2 d after inoculation. Co, conidium; Ap, appressorium; Ph, penetration hypha. Scale bar, 10 μm. (B) Percentage of normal appressoria and penetration hyphae formed on cellulose membranes. At least 300 appressoria on a membrane were observed at each experiment, and three independent experiments were performed. Values are the means of three replications; error bars represent ±SE. Bars with different letters indicate significant differences (Tukey’s test; *P* < 0.01). (C) Development of infection structures of the wild type and *cokel2* and *copag1* mutant strains on lower surface of detached cucumber cotyledons at 3 d after inoculation. Penetration hyphae were stained with lactophenol aniline blue. Scale bar, 10 μm. (D) Percentage of normal appressoria and penetration hyphae formed on lower surface of detached cucumber cotyledons. At least 300 appressoria on three cotyledons were observed at each experiment, and three independent experiments were performed. Values are means of three replications; error bars represent ±SE. Bars with different letters indicate significant differences (Tukey’s test; *P* < 0.01). (E) Pathogenicity assays on intact cucumber leaves after 7 d at 24°C. Six drops of a conidial suspension placed onto one half detached cucumber leaves; left side: wild type, right side: indicated mutants.

To define the function of CoPag1, we isolated *copag1* deletion mutants for the wild type and *cokel2Δ* strain. The gene replacements of *CoPAG1* were verified by DNA gel blot analysis (S3A and S3B Fig). To elucidate the function of CoPag1 on artificial substrates, we examined the *copag1* deletion mutants for the formation of appressoria and penetration hyphae on cellulose membranes (Fig 1A and 1B). Whereas *cokel2Δ* and *copag1Δ cokel2Δ* formed abnormal appressoria with lateral germination, *copag1Δ* produced normal appressoria similar to the wild type on artificial substrates and, moreover, retained its penetration ability on cellulose membranes (Fig 1A and 1B). These results demonstrated that CoPag1 is not involved in the CoKel2-dependent physical signaling pathway for appressorium morphogenesis. To define the involvement of CoPag1 in appressorium formation induced by plant-derived signals, we analyzed the phenotypes of *copag1* deletion mutants on the host plant. The *copag1Δ* and *cokel2Δ* mutants formed normal appressoria similar to the wild type on the plant surface (Fig 1C and 1D). By contrast, appressoria of *copag1Δ cokel2Δ* showed abnormal lateral germination on the host plant and did not form penetration hyphae (Fig 1C and 1D). Interestingly, abnormal appressoria formed by *copag1Δ cokel2Δ* strains developed from conidia that germinated bilaterally in contrast to *cokel2Δ* (Fig 1A and 1C), suggesting that *copag1* deletion leads to a defect in the determination of the germination site on the conidium. As expected, *copag1Δ cokel2Δ* was reduced in pathogenicity compared to single mutants (Fig 1E). These results clearly showed that *copag1Δ cokel2Δ* has defects both in the physical and plant-derived/chemical signal transduction pathway and that CoPag1 is a key component of the plant-derived signaling pathway for appressorium morphogenesis. To test the involvement of CoPag1 in appressorium formation on the other host leaf surface, conidial suspensions of the deletion strains were inoculated onto leaves of *Nicotiana benthamiana*. Whereas the *copag1Δ* and *cokel2Δ* mutants formed appressoria that were morphologically indistinguishable from those of the wild type, *copag1Δ cokel2Δ* produced abnormal appressoria (S4A and S4B Fig). Accordingly, leaves inoculated with *copag1Δ cokel2Δ* did not show disease symptoms in contrast to single mutants (S4C Fig), suggesting that the involvement of CoPag1 in the plant-derived signaling pathway was not specific to the cucumber leaf surface.

### NDR kinase Cbk1 plays an essential role for cell morphogenesis of infection structures and pathogenesis in *C*. *orbiculare*

MOR components, which include TAO3/MOR2/FRY-related scaffolding proteins, are highly conserved among fungi and higher eukaryotes [9, 14]. Therefore, we hypothesized that MOR plays a crucial role in appressorium development triggered by plant-derived signals in *C*. *orbiculare*. To test this hypothesis, we identified the NDR kinase *CoCBK1* (Cell-wall-Biosynthesis-Kinase-1), the homologous gene of *S*. *cerevisiae CBK1*, by searching the *C*. *orbiculare* genome database. The size of the predicted protein of CoCbk1 is 658 amino acids, and the catalytic domain of the fungal nuclear Dbf2-related kinase-like protein is conserved in CoCbk1 (S5 Fig).

**Fig 2:**
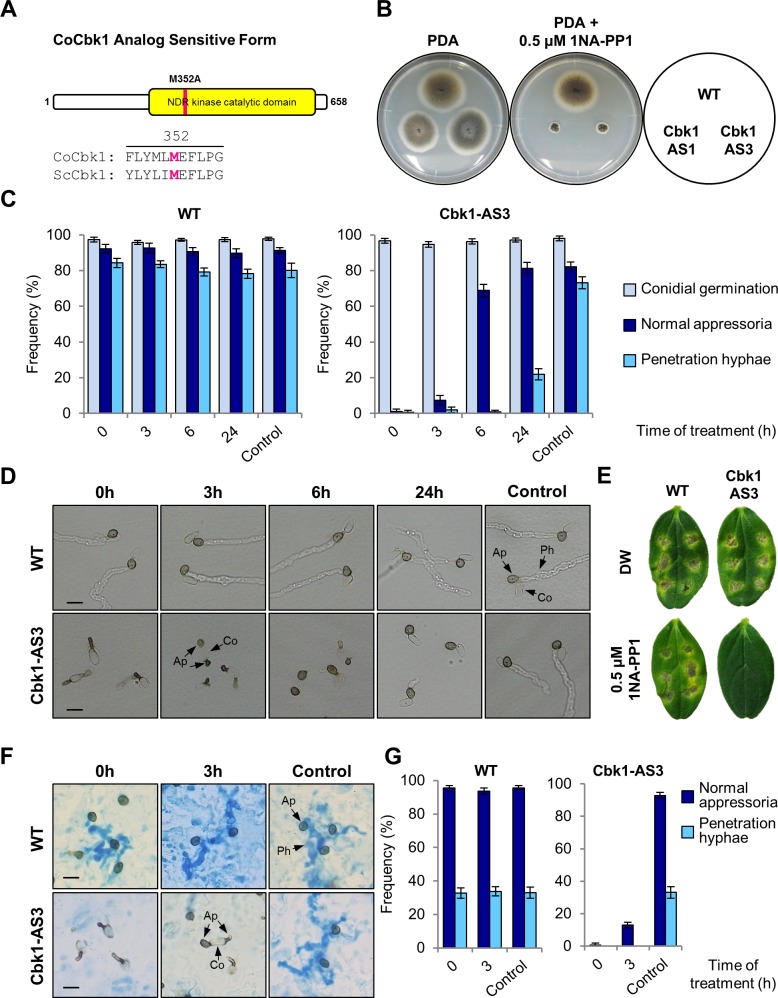
NDR kinase CoCbk1 plays an essential role for cell morphogenesis of infection structures and pathogenesis in *C*. *orbiculare*. (A) Schematic illustration of CoCbk1 analog-sensitive (AS) protein and sequence alignment including the ATP-binding pocket in *C*. *orbiculare* (Co) Cbk1 with corresponding sequences of *S*. *cerevisiae* (Sc) Cbk1. The kinase domain and site of the amino acid exchange (M352A) are marked. (B) Colonies of CoCbk1-AS strains on PDA and PDA supplemented with 0.5 μM 1NA-PP1. WT, wild type strain 104-T; Cbk1-AS1 and Cbk1-AS3, CoCbk1 analog-sensitive strain in WT background. (C) Mean percentage (±SE) of conidial germination, normal appressoria and penetration hyphae of CoCbk1-AS strains on cellulose membranes. Cells on a cellophane membrane were treated with 0.5 μM 1NA-PP1 at the indicated times. Cells were observed at 48 h after inoculation. At least 300 appressoria on a membrane were observed in each of three independent experiments. Values are means of three replications. Control, incubated in distilled water for 48 h. (D) Development of infection structures of CoCbk1-AS strains on cellulose membranes treated with 0.5 μM 1NA-PP1 at indicated times after start of incubation. Control, incubated in distilled water for 48 h; Co, conidium; Ap, appressorium; Ph, penetration hyphae. Scale bar, 10 μm. (E) Pathogenicity assay of CoCbk1-AS strains on intact cucumber cotyledons after 7 d at 24°C. Conidial suspensions of indicated strains were prepared in distilled water or in distilled water containing 0.5 μM 1NA-PP1 and dropped onto detached cucumber cotyledons. (F) Development of infection structures of CoCbk1-AS strains on lower surface of detached cucumber cotyledons treated with 0.5 μM 1NA-PP1 at indicated times after start of incubation. Cells were observed at 3 d after inoculation. Control, incubated in distilled water for 3 d; Scale bar, 10 μm. Penetration hyphae were stained with lactophenol aniline blue. (G) Mean percentage (±SE) of normal appressoria and penetration hyphae of CoCbk1-AS strains on lower surface of detached cucumber cotyledons. Cells were treated with 0.5 μM 1NA-PP1 at the indicated times and observed at 3 d after inoculation. At least 300 appressoria on three cotyledons were observed in each of three independent experiments. Values are means of three replications. Control, incubated in distilled water for 3 d.

We examined the effects of *cocbk1* targeted gene deletion on cellular morphogenesis. However, we were unable to generate *cocbk1Δ* deletion strains, due to apparent growth defects. To elucidate the function of CoCbk1 on cellular morphogenesis during the infection process of *C*. *orbiculare*, we generated an analog-sensitive CoCbk1^M352A^ variant (CoCbk1-AS) by site-directed mutagenesis (Fig 2A), which was fully active *in vivo* and was specifically inhibited by the ATP-analog 1NA-PP1 [21, 22]. On the basis of sequence alignments of CoCbk1 with *S*. *cerevisiae* Cbk1, we identified an M352 residue in the catalytic domain as the putative ATP-analog binding pocket (Fig 2A) [21]. CoCbk1-AS-replaced strains of the wild type were obtained and incubated on PDA media containing 1NA-PP1 for 7 days to test whether CoCbk1-AS strains are sensitive to 1NA-PP1. At 0.5 μM 1NA-PP1, mycelial growth of CoCbk1-AS strains was significantly inhibited (Fig 2B). By contrast, the inhibitor had no effect on the growth rate of the wild type. Therefore, strains expressing the CoCbk1-AS can be used to mimic the phenotype of CoCbk1 loss of function mutants. To investigate the role of CoCbk1 and functional relationship with CoPag1 in infection-related morphogenesis, we analyzed the development of appressoria and penetration hyphae on cellulose membranes and the host plant. Upon addition of 0.5 μM 1NA-PP1 during appressorium formation, the CoCbk1-AS strain was severely impaired in appressorium morphology, though conidial germination was not affected (Fig 2C, 2D, 2F and 2G). Intriguingly, addition of 1NA-PP1 at 3 h after inoculation, when conidia germinate, resulted in conidia that germinated bilaterally and formed abnormal appressoria (Fig 2D and 2F). This defect is similar to that observed in *copag1Δ cokel2Δ* mutants (Fig 1A and 1C), suggesting that CoCbk1 and CoPag1 have related functions during appressorium formation of *C*. *orbiculare*. To investigate the role of CoCbk1 in plant infection, we inoculated intact cucumber cotyledons with conidia of the wild type or the CoCbk1-AS strain and evaluated virulence 7 days after inoculation. The wild type caused disease symptoms in the presence of 0.5 μM 1NA-PP1, but the CoCbk1-AS strain did not (Fig 2E). Taken together, these results indicate that although CoCbk1 activity is essential for morphological differentiation of infection structures and pathogenesis, CoCbk1 has related function with CoPag1 in the determination of the germination site on the conidium.

### The MOR component Pag1 functions *via* activation of Cbk1 in *C*. *orbiculare*

**Fig 3:**
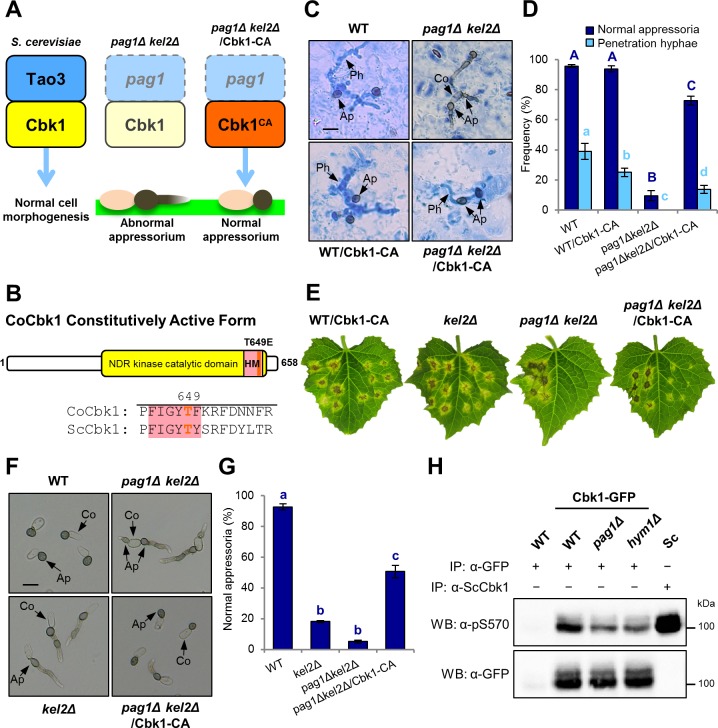
CoPag1 regulates the CoCbk1 activity. (A) Normal appressorium formation of *copag1Δ cokel2Δ* was restored by introducing the CoCbk1 constitutively active (CA) form. (B) Schematic drawing of CoCbk1 constitutively active (CA) protein and sequence alignment of *C*. *orbiculare* (Co) Cbk1 with corresponding sequences of *S*. *cerevisiae* (Sc) Cbk1. The kinase domain, the hydrophobic motif (HM) and the site of the amino acid exchange (T649E) are marked. (C) Infection structures of CoCbk1-CA strains on lower surface of detached cucumber cotyledons at 3 d after inoculation. Penetration hyphae were stained with lactophenol aniline blue. WT, wild type strain 104-T; *copag1Δ cokel2Δ*, *copag1* and *cokel2* double mutant strain; WT/ CoCbk1-CA, CoCbk1-CA strain in WT background; *copag1Δ cokel2Δ*/ CoCbk1-CA, CoCbk1-CA strain in *copag1Δ cokel2Δ* background. Co, conidium; Ap, appressorium; Ph, penetration hypha. Scale bar, 10 μm. (D) Mean percentage (±SE) of normal appressorium formation and penetration hyphal formation on lower surface of cucumber cotyledons. At least 300 appressoria on three cotyledons were observed in each of three independent experiments. Values are means of three replications. Bars with different letters indicate significant differences (Tukey’s test; *P* < 0.01). (E) Pathogenicity assay of CoCbk1-CA strains on intact cucumber leaves after 7 d at 24°C. Six drops of a conidial suspension was dropped onto one half of detached cucumber leaves; left sides: wild type; right sides: indicated mutant. (F) Appressorium development of CoCbk1-CA strains on petri dishes after 24 h at 24°C. Conidial suspensions of strains were prepared with distilled water. Scale bar, 10 μm. (G) Mean percentage (±SE) of normal appressorium formation of CoCbk1-CA strains on polystyrene petri dishes at 24 h after inoculation. At least 300 appressoria on a petri dish were observed in each of three independent experiments. Values are means of three replications. Bars with different letters indicate significant differences (Tukey’s test; *P* < 0.01). (H) CoCbk1 phosphorylation in *copag1Δ* and *cohym1Δ*. CoCbk1-GFP (102 kDa) was immunoprecipitated with anti-GFP from mycelia of the indicated strains. *S*. *cerevisiae* (Sc) Cbk1 (95 kDa) immunoprecipitated with anti-ScCbk1 was used as a positive control. The proteins were detected by western blotting using phosphospecific anti-pS570 (top) and anti-GFP (bottom).

In *S*. *cerevisiae*, Tao3 facilitates activation of the NDR kinase Cbk1, the central module in the RAM network [10]. Therefore, we wondered whether Pag1 functions *via* activation of Cbk1 in *C*. *orbiculare* (Fig 3A). To investigate this, we generated the constitutively active CoCbk1^T649E^ mutation (CoCbk1-CA) by site-directed mutagenesis (Fig 3B) and obtained the CoCbk1-CA strain in the *copag1* deletion background. *S*. *cerevisiae* Cbk1 works with five other RAM network proteins [10, 21]. In cells lacking any other RAM component, the C-terminal phosphorylation site Cbk1^T743^ residue located in a hydrophobic motif is not phosphorylated, and this phosphorylation is necessary for both cell separation and the maintenance of polarized growth [23]. Replacement of T743 with glutamic acid, which is known to mimic phosphorylation, bypassed the absence of a functional RAM network [24]. Expectedly, introducing CoCbk1-CA into *copag1Δ cokel2Δ* resulted in normal appressoria on the host surface in contrast to *copag1Δ cokel2Δ* (Fig 3C and 3D). Furthermore, pathogenicity of the *copag1Δ cokel2Δ*/ CoCbk1-CA strain was partially restored on the host plant compared with *copag1Δ cokel2Δ* (Fig 3E). These results suggest that CoPag1 functions *via* activation of CoCbk1 in *C*. *orbiculare*. In addition, normal appressorium formation of *copag1Δ cokel2Δ*/ CoCbk1-CA was restored on artificial substrates (Fig 3F and 3G), suggesting that constitutive activation of CoCbk1 provides normal morphogenesis of appressorium development in *copag1Δ cokel2Δ* without plant-derived signals. CoCbk1-CA introduction into the wild type resulted in normal appressoria but reduced virulence compared with the wild type (Fig 3C–3E). This result indicates that appropriate control of CoCbk1 activity is important for host infection in *C*. *orbiculare*.

Moreover, we found that deletion of *CoHYM1* (Hypha-like-Metulae-1), the homologous gene of RAM component *HYM1* in *S*. *cerevisiae* (S3C, S3D and S6A Figs), showed the defect in the determination of the germination site on the conidium similar to the CoCbk1-AS mutant (S6B and S6C Fig). By introducing CoCbk1-CA into *cohym1Δ*, normal appressorium formation was restored compared with the *cohym1Δ* (S6B and S6C Fig), indicating that CoHym1 is also involved in regulation of CoCbk1 activity in *C*. *orbiculare*.

To determine whether CoPag1 and CoHym1 regulate the phosphorylation of CoCbk1, we detected kinase activity of CoCbk1-GFP purified from the *copag1Δ* and *cohym1Δ* by western blotting using a phosphospecific antibody of *S*. *cerevisiae* Cbk1 [23]. Phosphorylation level of CoCbk1 in the *copag1Δ* and *cohym1Δ* was lower than that of the wild type (Fig 3H). In addition, in yeast two-hybrid assays, CoCbk1 interacted with CoPag1 and CoHym1 while no interaction was observed with CoKel2 (S7 Fig). These results indicate that CoPag1 and CoHym1 directly regulate the activity of CoCbk1 in *C*. *orbiculare*.

To test the specificity of the phenotype suppression by CoCbk1-CA, we analyzed the effect of CoCbk1-CA introduction into *cocac1* and *comekk1*- *cocmk1* mutants defective in of cAMP and MAP kinase signaling cascades, respectively in *C*. *orbiculare*. Conidia of the *cocac1*, *comekk1* and *cocmk1* mutants are defective in germination and development of appressoria on the surface of either the host plant or glass. In addition, these mutants are nonpathogenic to cucumber [1, 25–27]. Introduction of CoCbk1-CA into these mutants had no effect on these phenotypes (S8 Fig), suggesting that CoCbk1-CA specifically restores the morphological defect of mutants of MOR components, thus no direct link of MOR to cAMP and MAP kinase signaling cascade was recognized.

To characterize the constitutively active CoCbk1^T649E^ mutation on the transcript level, genome-wide transcriptional profiling of the CoCbk1-CA strain in the *copag1Δ* mutant background and the wild type strain 104-T during appressorium development *in vitro* was performed using custom microarrays with probes designed against the 13,479 *C*. *orbiculare* genes.

We collected samples from immature appressoria of each strain incubated on polystyrene petri dishes (*in vitro*) for 4 h. By 4 h, most conidia have germinated and started to develop appressoria. A total of 635 genes were differentially regulated (fold change >2, *P* < 0.05) in strain CoCbk1-CA compared with the wild type (S1 Data Set), suggesting that the constitutively active CoCbk1^T649E^ mutation causes transcriptional changes in *C*. *orbiculare* and that *CoCBK1* contributes to regulation of these genes during appressorium morphogenesis.

### Appressorium formation *via* Pag1 of *C*. *orbiculare* is induced by cutin monomers from the host plant cuticle

**Fig 4:**
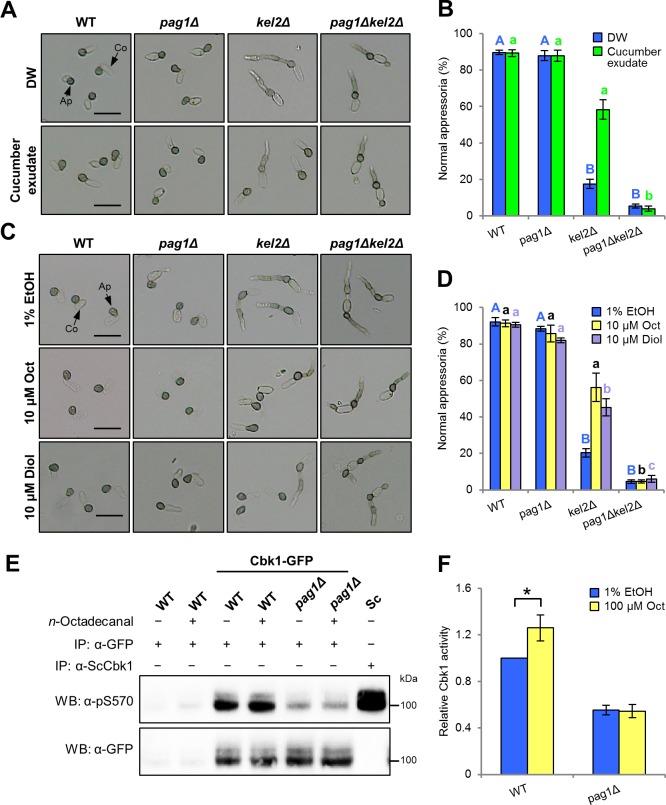
Cutin monomers from cucumber exudate function as a signal for inducing appressorium development of *C*. *orbiculare via* the MOR components. (A) Appressorium development in the presence of exudate from cucumber cotyledons. Conidial suspensions of indicated strains were prepared with distilled water or cucumber exudate and incubated on a petri dish at 24°C for 24 h. Co, conidium; Ap, appressorium. Scale bar, 20 μm. (B) Mean percentage (±SE) of normal appressorium formation in the presence of exudate from detached cucumber cotyledons. At least 300 appressoria on a polystyrene petri dish were observed at each experiment, and three independent experiments were performed. Values are means of three replications. Bars with different letters indicate significantly differences (Tukey’s test; *P* < 0.01). (C) Appressorium development in the presence of cutin monomers. Conidial suspensions of indicated strains were prepared in 1% ethanol (EtOH), 10 μM *n*-octadecanal (Oct) or 10 μM 1, 16-hexadecanediol (Diol) and incubated on a polystyrene petri dish at 24°C for 24 h. Scale bar, 20 μm. (D) Mean percentage (±SE) of normal appressorium formation in the presence of cutin monomers. At least 300 appressoria on a petri dish were observed in each of three independent experiments. Values are means of three replications. Bars with different letters indicate significant differences (Tukey’s test; *P* < 0.01). (E) CoCbk1 phosphorylation of CoCbk1-GFP purified from mycelia of the indicated strains, incubated in the presence of *n*-octadecanal. 4-day-old SD cultures were treated with 100 μM *n*-octadecanal (Oct) or 1% ethanol (EtOH) at 24°C for 24 h. The proteins were detected by western blotting using phosphospecific anti-pS570 (top) and anti-GFP (bottom). (F) Mean activity (±SE; *n* = 4) of CoCbk1-GFP purified from mycelia of the indicated strains, incubated in the presence of *n*-octadecanal. Protein levels are normalized to CoCbk1-GFP with anti-GFP antibody. The mean CoCbk1 activity of the wild type incubated in 1% EtOH was normalized to 1.0. Asterisk represents a significant difference (Student’s *t*-test; *P < 0.05).

To identify plant-derived signals responsible for induction of appressorium development mediated by Pag1 in *C*. *orbiculare*, we initially tested appressorium formation in the presence of cucumber exudate on a polystyrene petri dish. Normal appressorium formation of *cokel2Δ* was restored in the presence of cucumber exudate. By contrast, *copag1Δ cokel2Δ* formed abnormal appressoria when incubated in the presence of cucumber exudate similarly in distilled water (Fig 4A and 4B), suggesting that Pag1 of *C*. *orbiculare* is involved in controlling appressorium development upon recognition of chemical cue(s) present in the host. Because cutin monomers, a group of plant surface molecules, are known to trigger appressorium formation in other plant pathogenic fungi [28–30], we analyzed the effects of two cutin monomers on appressorium development through CoPag1. *n*-Octadecanal was isolated from cucumber exudate and 1, 16-hexadecanediol was used as a commonly used cutin monomer. While *cokel2Δ* developed normal appressoria in the presence of *n*-octadecanal or 1, 16-hexadecanediol on the petri dish, *copag1Δ cokel2Δ* formed abnormal appressoria either in the presence or absence of cutin monomers (Fig 4C and 4D). This result suggested that these cutin monomers functioned as a signal molecule for appressorium development *via* Pag1 in *C*. *orbiculare*.

To determine whether CoCbk1 is activated by cutin monomers, we tested the effect of *n*-octadecanal on the CoCbk1 phosphorylation. CoCbk1-GFP purified from mycelia of the wild type, incubated in the presence of *n*-octadecanal, exhibited the CoCbk1 phosphorylation 26% higher than that observed in the absence of *n*-octadecanal. By contrast, CoCbk1 phosphorylation of *copag1Δ* was not affected by *n*-octadecanal (Fig 4E and 4F). Thus, it indicated that *n*-octadecanal activates CoCbk1 dependent on CoPag1.

**Fig 5:**
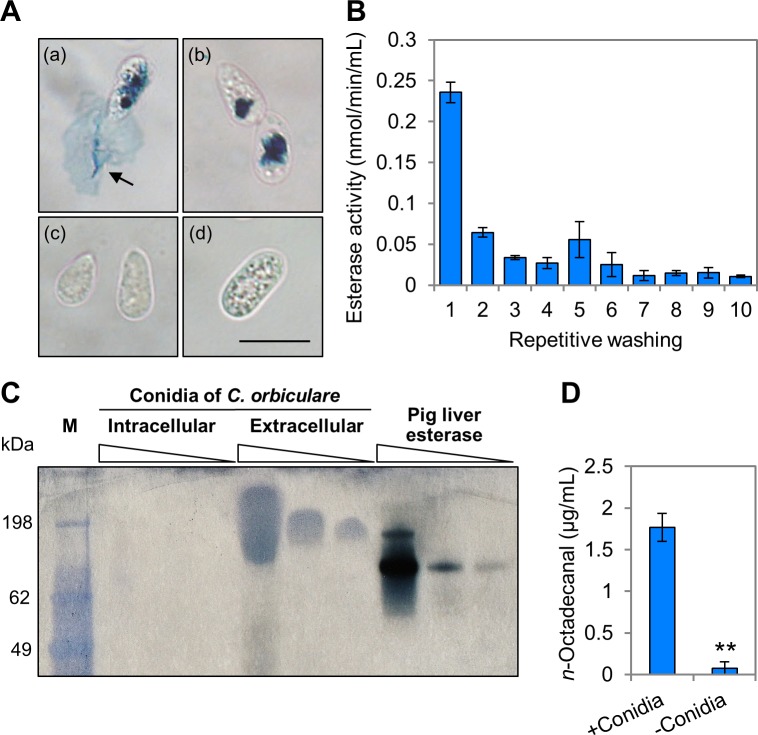
Extracellular esterases from the surface of *C*. *orbiculare* conidia hydrolyze the host plant cuticle. (A) Histochemical demonstration of surface-localized esterase activity using indoxyl acetate as a substrate. (a) Unwashed conidia were moved from the adhesion surface and underlying indigo blue crystals were exposed (arrow). (b) No crystals appeared on the adhesion surface of washed conidia. (c) Autoclaved conidia had no indigo blue crystals. (d) Unwashed native conidium on medium without indoxyl acetate. Scale bar, 10 μm. (B) Mean activity (±SE) of esterase released from surface of conidia by repetitive washing. Activity in conidial washing supernatants was evaluated with *p*-nitrophenyl butyrate as the substrate. Values are means of three replications. (C) Conidial surface esterases of *C*. *orbiculare* in native polyacrylamide gel after electrophoresis. Intracellular, protein preparations isolated from washed conidia; extracellular, protein preparations washed from conidia; pig liver esterase, a positive control of hydrolysis; M, molecular weight marker. Amount of protein loaded was (left to right) 1.0, 0.1 and 0.05 μg. (D) Quantity of *n*-octadecanal in cucumber exudate (means ± SE; *n* = 3). *n*-Octadecanal was detected in the exudate collected with conidial suspension (+conidia), but not in the exudate collected with distilled water (-conidia). Asterisk represents a significant difference (Student’s *t-*test; **P < 0.01).

In rust fungus *Uromyces viciae-fabae* and the powdery mildew fungus *Blumeria graminis*, extracellular cutinase and esterase from the surface of spores assist in the adhesion to the host cuticle and initiation of the infection process [3]. Therefore, we hypothesized that cutinase and esterase released from conidia of *C*. *orbiculare* hydrolyze the plant surface cuticle to form cutin monomers, and these cutin monomers induce appressorium development *via* MOR. To test whether hydrolytic enzymes are released from the conidia, conidia were placed on glass slides coated with an assay medium containing indoxyl acetate as the esterase substrate. Indeed, indigo blue crystals formed on the adhesion site of unwashed conidia (Fig 5A). By contrast, no crystals appeared on the adhesion surface of washed conidia that had surface enzymes removed, and autoclaved conidia failed to accumulate crystals either intracellularly or extracellularly (Fig 5A). These results indicate that enzyme activity is localized on the conidial surface in *C*. *orbiculare*. Additionally, esterase activity released from conidia was evaluated with *p*-nitrophenyl butyrate as the substrate. Conidia were repeatedly washed using a vortex, and each washing supernatant was assayed for esterase activity. The first washing supernatant had the highest activity, and subsequent washings had rapidly less enzyme activity than each previous wash (Fig 5B), suggesting that the release of esterase occurred rapidly on contact with an aqueous environment. The localization of esterase on the conidial surface was also shown by native polyacrylamide gel electrophoresis (Fig 5C). Whereas esterases were not detected in the intracellular protein preparation, one band of the extracellular esterase was visualized in the indoxyl acetate assay.

To investigate that conidial surface esterases are involved in the release of cutin monomers from the leaf surface, we measured the quantity of *n*-octadecanal in the cucumber exudate by GC-MS (Fig 5D). Droplets of either conidial suspension or distilled water were placed on the surface of cotyledons for 1 h at 24°C, then collected. Intriguingly, *n*-octadecanal was detected only in the cucumber exudate collected with the conidial suspension (Fig 5D), indicating that conidial surface esterases hydrolyze plant surface cuticle to cutin monomers; the concentration of *n*-octadecanal in the cucumber exudate collected with the conidial suspension, 1.8 μg/mL (6.7 μM), is a concentration sufficient for inducing normal appressorium development of *cokel2Δ* (Fig 4C and 4D). Taken together, we conclude that appressorium development *via* enhanced activation of CoCbk1 is induced by cutin monomers derived from hydrolysis of the host plant cuticle by conidial surface esterases.

### The MOR of *C*. *orbiculare* is responsible for the expression of plant-signal-induced genes

**Fig 6:**
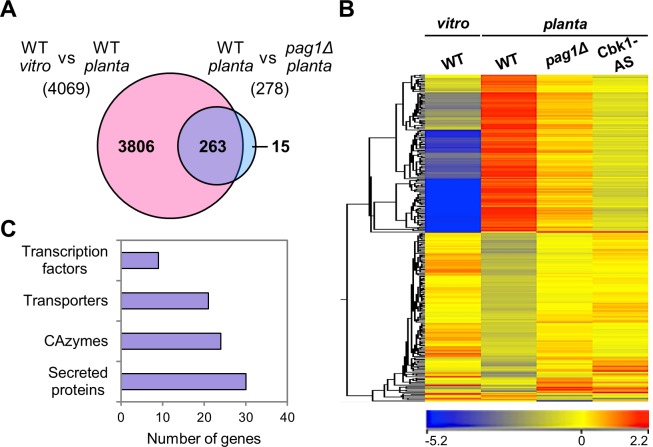
The MOR components of *C*. *orbiculare* are responsible for the expression of the plant-signal-induced genes. (A) Venn diagram illustrating overlaps between number of upregulated genes (red) during appressorium formation *in planta* compared with *in vitro* and downregulated genes (blue) in *copag1Δ* compared with the wild type, both incubated *in planta*. (B) Hierarchical clustering heatmap of gene expression showing the differentially regulated genes in *copag1Δ* compared with the wild type, both incubated *in planta*. For inhibiting of CoCbk1 activity of CoCbk1-AS, 0.5 μM 1NA-PP1 was applied at 3 h after inoculation. Differential expression of each gene is indicated in color (red: induced, blue: repressed; numbers below scale: fold change). (C) Number of plant-signal-induced genes that were downregulated in *copag1Δ* compared with the wild type, both incubated *in planta*, in four functional categories.

To examine the involvement of CoPag1 and CoCbk1 in regulation of genes that are specifically expressed during appressorium development *in planta*, we analyzed the transcriptome at the stage of appressorium formation *in vitro* and *in planta* using custom microarrays. We collected samples from immature appressoria of the wild type strain 104-T after incubating for 4 h on petri dishes (*in vitro*) and from the wild type, *copag1Δ* and CoCbk1-AS treated with 1NA-PP1 after 4 h on cucumber cotyledons (*in planta*). In the wild type strain, 4069 genes (30%) were upregulated *in planta* compared with *in vitro* (fold change >2, *P* < 0.05), indicating that the transcriptome of *C*. *orbiculare* was highly responsive to plant-derived signals during appressorium development (Fig 6A). In a hierarchical clustering of the 550 genes that are differentially regulated in *copag1Δ* compared with the wild type incubated *in planta*, 278 genes were downregulated in *copag1Δ* compared with the wild type *in planta* (Fig 6B). Furthermore, the 278 genes were downregulated in CoCbk1-AS mutant treated with 0.5 μM 1NA-PP1 (Fig 6B), indicating that *CoPAG1*-dependently expressed genes were under control of *CoCBK1* in *C*. *orbiculare*. Of these 278 genes, 263 (95%) were plant-signal-induced genes, suggesting that *CoPAG1* specifically contributes to the expression of the plant-signal-induced genes (Fig 6A). Interestingly, among the 263 genes, 30 encode secreted proteins including 16 small secreted proteins (SSPs; predicted length <300 amino acids), 24 encode carbohydrate-active enzymes (CAzymes) including 12 enzymes associated with the degradation of plant cell wall constituents, 21 encode transporters and 9 transcription factors (Fig 6C). CAZymes [31] that potentially degrade the plant cell wall [32] and remodel the fungal cell wall are thus important for establishment of infection. In addition, fungal-secreted proteins including effectors that facilitate infection by manipulation of host metabolism and evasion of host immunity [33]. Concomitantly, plasma membrane transporters function in the secretion of protein effectors and secondary metabolites including toxins to the plant cell or protection against plant defense compounds or disease control agents [34, 35]. Therefore, the MOR components seemed to be linked to expression of a subset of the infection-related genes.

## Discussion

### *C*. *orbiculare* Pag1, a component of the MOR, is crucial for appressorium development triggered by plant-derived signals

**Fig 7:**
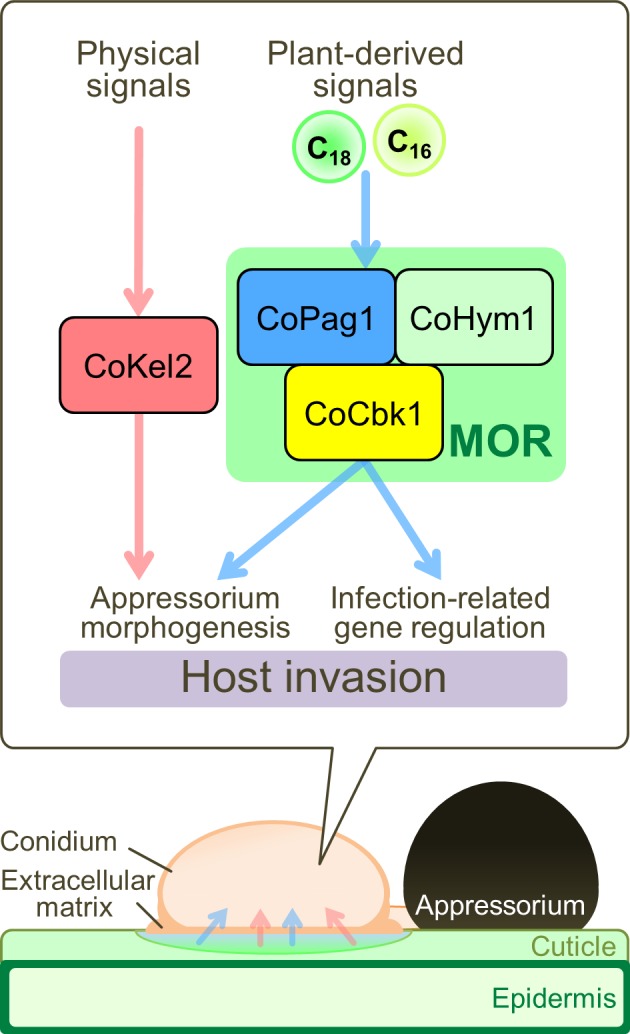
Model for appressorium-mediated invasion of *C*. *orbiculare* in response to plant-derived signals. Esterase activity in the extracellular matrix of conidia frees the cutin monomer *n*-octadecanal (C_18_) from plant surface cutin to serve as the signal molecule for appressorium development *via* the MOR components of *C*. *orbiculare*. 1,16-Hexadecanediol (C_16_) also induces appressorium development mediated by CoPag1. After perception of the plant-derived signals, enhanced activation of CoCbk1 dependent on CoPag1 is required for appressorium morphogenesis independent of CoKel2 function and contributes to the regulation of infection-related genes by communicating with plant-derived signals.

Appressorium development is driven by the perception of one or several host-derived signals. Previous work on *U*. *maydis* demonstrated that hydrophobicity and hydroxy fatty acids/cutin monomers stimulate the differentiation of appressoria [30]. Furthermore, two plasma membrane proteins, Sho1 and Msb2, that act upstream of MAP kinases Kpp2 and Kpp6, have been suggested to be involved in sensing of the hydrophobic surface for appressorium formation in *U*. *maydis* [36]. In *M*. *oryzae*, MoSho1 and MoMsb2 regulate surface recognition for appressorium formation *via* MAP kinase signaling [37]. In addition, Pth11, a putative G protein-coupled receptor (GPCR) is involved in host surface recognition acting upstream of the cAMP pathway [38]. Previously, we elucidated that *CoKEL2*, a *S*. *pombe tea1* homolog is required for appressorium development on artificial surfaces [7]. The *cokel2Δ* mutant displayed abnormal appressoria, germinated laterally, and was defective in penetration into cellulose membranes (Fig 1A and 1B). Surprisingly however, the *cokel2Δ* mutant was normal in appressorium formation and penetration on the host plant surface (Fig 1C and 1D). Therefore, we hypothesized that CoKel2 is important for recognizing physical signals but not chemical signals, and a bypass mechanism was induced by chemical signals from the host plant, independent of CoKel2 function. Whereas plant pathogenic fungi including *C*. *orbiculare* have developed different mechanisms regulating appressorial development such as cAMP/PKA and MAP kinase signaling pathways, the signal transduction pathway specifically activated by plant-derived signals is unknown. In this study, we identified CoPag1 as a key component of the signaling cascade for appressorium formation activated by cutin monomers from the plant surface (Figs 1 and 4, S1 Table). Genome-wide transcriptional profiling showed that 95% of the CoPag1-dependently expressed genes were plant-signal-induced genes (Fig 6A), suggesting that CoPag1 plays a crucial role in the signal transduction pathway for sensing and responding to plant-derived cues in *C*. *orbiculare*. On the other hand, the normal appressorium formation of *copag1Δ* on artificial surfaces suggests that CoPag1 is not involved in the CoKel2-dependent physical signal transduction pathway (Fig 1A and 1B, S1 Table). If CoPag1 is not specific to chemical signals but involved in physical signals, the *copag1Δ* mutant would not form normal appressoria on artificial surfaces. Therefore, we propose that two separate cascades, Kel2- and Pag1-dependent, are induced by the physical and chemical signals, respectively, and function redundantly in appressorium development of *C*. *orbiculare* (Fig 7). Consistent with this hypothesis, the transcriptome of *C*. *orbiculare in planta* differed from that *in vitro* at the stage of appressorium development, although the appressoria on the two surfaces are morphologically indistinguishable (Fig 6A). In addition, introduction of the phospho-mimetic point mutation did neither affect cAMP nor MAP kinase signaling pathway mutant phenotype (S8 Fig), indicating the independency of the MOR from those signaling pathways.

In this study, out of six insertional mutants, we identified a tagged gene besides *CoPAG1*. The mutated gene of PDM-7 showed significant homology to *S*. *cerevisiae SNF1* (Sucrose-Non-Fermenting-1) kinase gene. Because of the other four mutants were not tagged by T-DNA insertion, we tested whether these phenotypes were caused by disruption of *CoPAG1*. Whereas introduction of *CoPAG1* to PDM-4 restored normal appressorium formation and pathogenicity, phenotypes of *CoPAG1* complemented strains in the other PDMs were not affected (S1A–S1E Fig), indicating that the disrupted genes of these PDMs were not *CoPAG1*. In addition, we examined whether these mutations are under control of CoCbk1 by introducing CoCbk1-CA to PDMs and evaluation of their phenotype (S1C–S1E Fig). This provided a possibility that these mutations lie up- or down-stream of CoCbk1 shown in S1F Fig.

### The MOR coordinates plant-derived signals with polarized morphogenesis of appressoria and pathogenesis in *C*. *orbiculare*

NDR kinases are regulated by phosphorylation at two conserved sites [8]. A serine residue within the kinase domain is autophosphorylated for the basal kinase activity [9, 14]. Activation further requires a second phosphorylation in the C-terminal hydrophobic motif of the kinase [9, 14]. In *C*. *orbiculare*, the introduction of phospho-mimetic point mutations in the hydrophobic motif of CoCbk1 (T649E) suppressed the defective phenotype of *copag1* and *cohym1* mutants (Fig 3 and S6 Fig). Moreover, the level of CoCbk1 phosphorylation at the autophosphorylated serine residue (S477) of *copag1Δ* and *cohym1Δ* was lower than that of the wild type (Fig 3H). Yeast two-hybrid assays revealed that CoCbk1 interacted with CoPag1 and CoHym1 (S7 Fig). These results indicate that CoPag1 and CoHym1 directly regulate the CoCbk1 activity through its phosphorylation at the hydrophobic motif and at the autophosphorylated serine residue of the kinase domain. Moreover, CoCbk1 is further activated by the cutin monomer dependent on CoPag1 (Fig 4E and 4F). The *copag1Δ cokel2Δ* formed abnormal appressoria in the presence of cutin monomers compared with *cokel2Δ* (Fig 4C and 4D). Therefore, we propose that enhanced activation of CoCbk1 dependent on CoPag1 required for appressorium development responding to cutin monomers in *C*. *orbiculare*.

The MOR regulates cell polarity and morphogenesis in fungi. Despite the high conservation of its components, deletion phenotypes of MOR components in various fungi are diverse, ranging from loss of polarity to hyperpolarization and excessive branch formation. In *C*. *orbiculare*, gene deletion mutants of *CoCBK1* and the uncharacterized MOR components (*CoMOB2*, *CoKIC1*, *CoSOG2*) could not be obtained due to mycelial growth defect, whereas *copag1* and *cohym1* deleted mutants were obtained and did not show growth defectiveness. In western blotting with phosphospecific antibody, *copag1* and *cohym1* mutants retained partial CoCbk1 activity (Fig 3H). Thus, the difference in the deletion phenotypes seems in consistent with the CoCbk1 phosphorylation level. To evaluate the role of CoCbk1 in appressorium development, we used the CoCbk1-AS mutant. The conditional inactivation of CoCbk1 and *cohym1* deletion resulted in abnormal appressoria from bilateral germination of the conidia (Fig 2D and 2E, S6B Fig). Similarly, *copag1Δ cokel2Δ* is defective in the determination of conidial germination site, although *copag1Δ* is normal in appressorium development because the physical signal pathway mediated by CoKel2 functions redundantly (Fig 1A and 1C). These results indicate that the lower CoCbk1 activity leads to a defect in polarized morphogenesis during conidial germination in *C*. *orbiculare*. On the other hand, constitutively active CoCbk1 mutation in the wild type resulted in morphologically normal appressorium formation, but its virulence was slightly lower than that of the wild type (Fig 3E). Therefore, proper regulation of the CoCbk1 activity is required for appressorium-mediated infection of *C*. *orbiculare*.

One of the open questions in research on MOR concerns the identification of upstream activators and downstream targets of the cascade. In *C*. *orbiculare*, the MOR components CoPag1 and CoCbk1 is involved in appressorium development induced by cutin monomers, suggesting that MOR of *C*. *orbiculare* at least indirectly links to an upstream receptor for cutin monomers. There is no report about a sensor for cutin monomers in fungi, but G-protein-coupled receptors (GPCRs) activated by free fatty acids have been identified in mammals [39]. Thus, GPCRs have the potential to sense cutin monomers. In *M*. *oryzae*, 76 GPCR-like receptors were found, including 61 Pth11-like proteins [40]. Further research on GPCRs could help us understand the relation between MOR and extracellular signal sensing in fungi. On the other hand, from our genome-wide gene expression analysis, we found that *CoPAG1* contributes to regulation of a subset of the plant-signal-induced genes predicted to encode secreted proteins, CAzymes, transporters and transcription factors (Fig 6). These genes were under control of *CoCBK1* (Fig 6B). Although a virulence function of these genes has not yet been demonstrated, it is tempting to suggest that these genes contribute to infection of *C*. *orbiculare*. Consistent with this hypothesis, the *copag1Δ* formed morphologically normal appressoria, but virulence was slightly lower than that of the wild type (Fig 1). Therefore, the MOR components are responsible for the expression of a subset of the plant-signal-induced genes with potential roles in pathogenicity of *C*. *orbiculare*.

### The cutin monomer degraded from the host plant cuticle functioned as a signal molecule for appressorium development *via* CoCbk1 activation by CoPag1

Cutin monomers are well-known surface signals recognized by several fungal plant pathogens [28–30]. *n*-Octadecanal, identified in the exudate from cucumber leaves, and 1, 16-hexadecanediol induced normal appressorium formation of *cokel2Δ* but not of *copag1Δ cokel2Δ* (Fig 4C and 4D). Using biochemical approaches, we showed that *n*-octadecanal promotes CoCbk1 activation in the wild type but not in *copag1Δ* (Fig 4E and 4F), implying that the CoCbk1 is activated by the cutin monomer mediated by CoPag1 in *C*. *orbiculare*. These results indicate that cutin monomers function as key signal molecules for appressorium formation through MOR components in *C*. *orbiculare*. In addition, appressorium morphogenesis of *copag1* and *cokel2* mutants on *N*. *benthamiana*, a susceptible host plant for *C*. *orbiculare* showed no distinctive difference to that on cucumber leaves (S4 Fig), suggesting that appressorium formation is mainly responding to cutin monomers common in host plants.

The extracellular matrix that surrounds spores plays a role in attachment of the host surface and the pre-penetration development of many plant pathogens [3]. Thus, we hypothesized that conidial surface esterases may be involved in the release of cutin monomers from the leaf surface. Previous studies revealed that the extracellular matrix of *Colletotrichum* species contains high molecular weight mannose-rich glycoproteins, germination inhibitors and a variety of enzymes, including cutinase [41]. Consistent with this, esterase activity is localized on the conidial surface in *C*. *orbiculare* (Fig 5A–5C). The release of esterase occurred rapidly on contact with an aqueous environment (Fig 5B). Most notably, *n*-octadecanal was detected in the cucumber exudate collected with the conidial suspension, but not in the exudate collected with distilled water (Fig 5D), indicating that conidial surface esterases hydrolyze plant surface cuticle to cutin monomers. The amount of *n*-octadecanal in the exudate with the conidial suspension was reasonable for inducing normal appressorium formation by *cokel2Δ* (Figs 4C, 4D and 5D). Taken together, these data suggest that conidial surface esterases hydrolyze the cuticle to generate the signal molecule for appressorium development *via* the MOR components of *C*. *orbiculare* (Fig 7).

## Materials and methods

### Strains and growth conditions

Strain 104-T (MAFF240422) of *C*. *orbiculare* (Berk. & Mont.) Arx [syn. *C*. *lagenarium* (Pass.) Ellis & Halst.] was used as the wild type strain [42]. All *C*. *orbiculare* strains were maintained at 24°C in the dark on 3.9% (w/v) PDA (Difco) or SD medium (0.67% w/v yeast nitrogen base without amino acids, 2% w/v glucose, 2% w/v agar). *E*. *coli* DH5α competent cells were used as the host for gene manipulation and maintained on Luria-Bertani medium [43] at 37°C. *Agrobacterium tumefaciens* strain C58C1 was used as the T-DNA donor for fungal transformation and maintained on LB medium at 28°C.

### Pathogenicity tests and infection structure differentiation

The inoculation assays on detached cucumber leaves (*Cucumis sativus* L. ‘Suyo’) were performed as described previously [44]. Conidia of *C*. *orbiculare* were obtained from 7-day-old PDA cultures, and six drops of 10 μL of a conidial suspension (5 × 10^5^ conidia/mL) were placed on the surface of cucumber leaves. The leaves were incubated in a humid box at 24°C with a 16 h photoperiod for 7 days.

For appressorium formation and penetration assays *in vitro*, 20 μL droplets or 1 mL of a conidial suspension (5 × 10^5^ conidia/mL distilled water) was placed on a polystyrene petri dish or a cellophane membrane (Wako Chemicals), respectively, and incubated in a humid environment at 24°C in the dark. For penetration assays *in planta*, 10 μL of a conidial suspension (5 × 10^5^ conidia/mL distilled water) was spotted onto the abaxial surface of cucumber cotyledons and incubated in a humid box at 24°C. After 3 days, the lower epidermis of the cotyledons were peeled off and stained with 0.1% lactophenol aniline blue solution [45].

### Fungal transformation

The *Agrobacterium tumefaciens*-mediated transformation (AtMT) protocol was applied with slight modifications of a previously described method [44]. Hygromycin-resistant transformants were selected on PDA containing 100 μg/mL hygromycin B (Wako Chemicals), 25 μg/mL meropenem hydrate (Sumitomo Dainippon Pharma). Bialaphos-resistant transformants were selected on SD medium containing 4 μg/mL bialaphos (Meiji Seika Kaisha) and 25 μg/mL meropenem hydrate. Sulfonylurea-resistant transformants were selected on SD medium containing 4 μg/mL chlorimuron ethyl (Maruwa Biochemical) and 25 μg/mL meropenem hydrate. All *C*. *orbiculare* strains generated in this study are listed in S2 Table.

### Identification of candidate-mutated genes of insertional mutants in *cokel2Δ* background by whole genome sequence analyses

The 10,021 insertional mutants in the *cokel2Δ* background were screened using the AtMT protocol as described previously [25]. Whole genome sequences of six insertional mutants (PDM-2, PDM-3, PDM-4, PDM-5, PDM-6 and PDM-7) were investigated using Illumina HiSeq 2000. T-DNA insertion sites were detected by comparison with the wild type strain 104-T genome sequence.

### Molecular phylogenetic analyses

Multiple sequence alignments were performed using the Clustal W program [46]. Phylogenetic trees were constructed using MEGA 6 (www.megasoftware.net) [47] with the minimum-evolution method, based on 1000 replicates. The amino acid sequences of Pag1 homologs were obtained from the database of the National Center for Biotechnology Information (http://www.ncbi.nlm.nih.gov/).

### Complementation of the *S*. *cerevisiae tao3* mutant with *C*. *orbiculare PAG1* cDNA

For synthesizing *CoPAG1* cDNA, total RNA was extracted from vegetative mycelium of *C*. *orbiculare* by using RNeasy Plant Mini Kit (Qiagen). *CoPAG1* cDNA was amplified by RT-PCR using primer pairs CoPAG1orf_F3/CoPAG1orf_R2 and CoPAG1orf_F2/CoPAG1orf_R1. The amplified PCR products were introduced into the yeast cDNA expression vector pYES2 (Invitrogen) using the In-Fusion HD cloning kit (Clontech). These primers are listed in S4 Table. As a positive control, a plasmid expressing *S*. *cerevisiae TAO3* was also constructed. The genomic DNA of *TAO3* was amplified by PCR using primer pair ScTAO3_F1/ScTAO3_R1. Yeast cells were transformed using the lithium acetate method [48]. Yeast cells were then grown on yeast SC minimal medium agar lacking uracil (U). For observation of yeast cell morphogenesis, cells were cultured overnight either in SC or SC-U containing 20% galactose and 10% raffinose at 30°C. The axial ratio (length/width) of cells was calculated from images analyzed with the software ImageJ (National Institutes of Health, http://rsb.info.nih.gov/ij/).

### Plasmid construction

All plasmids were derived from a binary vector pBIG4MRBrev carrying the bialaphos-resistance gene cassette and pBIG4MRSrev carrying the sulfonylurea-resistance gene cassette. For plasmid constructions, the In-Fusion HD Cloning Kit (Clontech) or GENEART Seamless Cloning and Assembly Kit (Thermo Fisher Scientific) were used. All primers used in this study are listed in S4 Table.

For generating the *copag1* disruption vector, 1.0-kb upstream and downstream flanking sequences were amplified from *C*. *orbiculare* genomic DNA, and a 1.4-kb fragment of the hygromycin-resistance gene was amplified from pBIG4MRHrev with the respective primers (S4 Table). These three fragments were inserted into a linearized pBIG4MRSrev. The same procedure was applied to generate the *cohym1* disruption vector.

For generating an analog-sensitive CoCbk1^M352A^ variant (ATG > GCG), a 2.3-kb fragment containing the 5′-region of *CoCBK1* and a 2.0-kb fragment containing the 3′-region of *CoCBK1* were amplified from *C*. *orbiculare* genomic DNA with the respective primers to generate a point mutation (S4 Table). These two fragments were inserted into pBIG4MRBrev. The hygromycin-resistance gene or the sulfonylurea-resistance gene were amplified with the respective primers (S4 Table) and inserted downstream of the 3′ untranslated region of *CoCBK1* in the CoCbk1^M352A^ point mutation plasmid pBI-CoCbk1-asB.

For generating a constitutively active CoCbk1^T649E^ variant (ACA > GAG), a 3.2-kb fragment containing the 5′-region of *CoCBK1* and a 3.8-kb fragment containing the 3′-region of *CoCBK1* and the sulfonylurea-resistance gene inserted downstream of the 3′ flanking region of *CoCBK1* were amplified from the CoCbk1^M352A^ point mutation plasmid pBI-CoCbk1-asBS with the respective primers (S4 Table). These two fragments were inserted into pBIG4MRBrev.

For constructing the *CoCBK1-GFP* fusion gene vector regulated by the *CoCBK1* native promoter, a *CoCBK1* fragment containing its 1.2-kb upstream and 900-bp downstream flanking sequences were amplified from *C*. *orbiculare* genomic DNA. The fragment was inserted into a linearized pBIG4MRBrev. The GFP fragment was amplified from pBI-glyGFP and inserted at the C-terminal end of *CoCBK1* in the *CoCBK1* complementation plasmid pBI-CoCBK1-B.

### Yeast two-hybrid assays

The yeast two-hybrid screen was performed using the instructions of the Matchmaker Gold Yeast Two-Hybrid System (Clontech). Full-length cDNAs of putative interaction partners were generated from *C*. *orbiculare* mycelia. The genes encoding the proteins tested for interaction were cloned into the pGBKT7 or pGADT7 vectors (Clontech) to express fusion proteins with the yeast GAL4 binding (BD) and activation domain (AD), respectively. All BD or AD constructs were used to transform the Gold or Y187 yeast strain, respectively (Clontech). After mating, diploid yeast was plated on double dropout synthetic selective medium lacking Trp and Leu (DDO) for mating control and on stringent medium supplemented with 20 mg/mL X-α-Gal and 100 ng/mL aureobasidin A (DDO/X/A) and incubated at 30°C for 5 d. Protein interactions were assessed by growth of diploid yeast on DDO, DDO/X/A, and stringent quadruple dropout synthetic selective medium lacking Trp, Leu, Ade and His (QDO) compared with corresponding controls. All yeast strains generated in this study are listed in S3 Table.

### Genomic DNA gel blot analysis

Genomic DNA of *C*. *orbiculare* was isolated from mycelia, and DNA blot analysis was done by standard methods. DNA probes were labeled with DIG-dUTP using the *Bca*BEST DIG Labeling Kit (Takara Bio). Hybridized DNA was detected using Anti-Digoxygenin-AP Fab fragments (Roche Diagnostics), and light emission generated by enzymatic dephosphorylation of CDP-Star Detection Reagent (GE Healthcare) by alkaline phosphatase was detected using the FUJIFILM LAS1000 plus gel documentation system (Fujifilm).

### Chemical treatments

The CoCbk1 activity of an analog-sensitive CoCbk1^M352A^ variant (CoCbk1-AS) was inhibited by adding 0.5 μM 1NA-PP1 (Carbosynth Ltd.). For pathogenicity tests, conidia were suspended in 0.5 μM 1NA-PP1 at 5 × 10^5^ conidia/mL. For penetration assays *in vitro*, 0.5 μM 1NA-PP1 was applied to cells on a cellophane membrane at the appropriate time.

For collecting exudates from cucumber cotyledons, 10 μL droplets of the wild type conidial suspension (5 × 10^5^ conidia/mL distilled water) were placed on the surface of cotyledons for 1 h at 24°C, then collected by filtering through a 0.2-μm-pore filter (Sartorius). For testing the effects of cutin monomers on appressorium development, stock solutions of 1 mM *n*-octadecanal and 1mM 1, 16-hexadecanediol were prepared in 100% ethanol, then diluted with distilled water to 10 μM. Conidia were suspended to 5 × 10^5^ conidia/mL in the cucumber exudate, 10 μM *n*-octadecanal, 10 μM 1, 16-hexadecanediol, distilled water or 1% ethanol as the control experiments.

### Isolation of cutin monomer *n*-octadecanal from cucumber leaves and quantitative analysis by GC-MS

Drops of water applied to the leaf surface were collected (total 3~5 mL) and lyophilized. The dry residue was dissolved in methanol (100 μL) and directly analyzed by GC-MS (Shimadzu GCMS-QP5050A) using a TC-FFAP capillary column (30 m × 0.25 mm). The GC was temperature programmed with an initial 1 min at 200°C, then a rise of 10°C/min to final isothermal period at 280°C. Detector temperature was set at 250°C. For quantitative determination, standard *n*-octadecanal was synthesized from commercially available *n*-octadecanoic acid (Wako Chemicals) by reduction with diisobutylaluminum hydride. The structure was confirmed by H-NMR (Bruker AVANCE III 400) and ESI-MS (Shimadzu LCMS-8040).

### Protein purification and western blotting with phosphospecific antibodies

All mycelial samples were prepared from 5-day culture at 24°C in SD broth. For the cutin monomer induction assay, 100 μM *n*-octadecanal was applied at 4-day culture and incubated further 24 h. The protein lysates for immunoprecipitation were prepared from mycelia by grinding in liquid nitrogen. The tissue powder was dissolved in the lysis buffer contained 50 mM Tris-HCl, pH 7.4, 150 mM NaCl, 10% glycerol, 0.1% v/v Triton X-100, 1mM DTT, 60 mM β-glycerophosphate, 10 mM NaF, 1 mM sodium molybdate, 0.1% cantharidin and a protease inhibitor cocktail tablet (Roche Diagnostics), and total extracts were clarified by centrifugation at 16,900 × *g*, 4°C, 20 min. The CoCbk1-GFP proteins were immunoprecipitated from total protein extracts using anti-GFP (abcam) coupled to recombinant Protein G Sepharose beads (Thermo Fisher Scientific) as described [49].

Western blotting with phosphospecific antibodies [22] was modified from [49]. Proteins were separated by SDS-PAGE using a Novex 3–8% Tris-Acetate gel (Thermo Fisher Scientific) and transferred to a PVDF membrane (Thermo Fisher Scientific). Membranes were blocked for 1 h at 24°C with 5% skim milk in Tris-buffered saline with 0.1% Tween (TBST). The primary antibodies were diluted 1:1,000 (anti-pS570) or 1:2,500 (anti-GFP) in TBST and incubated 1 h at 24°C. After washing three times for 5 min with TBST, membranes were then incubated with HRP-conjugated anti-rabbit secondary (Vector Laboratories) at 1:1,000 in TBST with 5% skim milk for 1 h at 24°C. Membranes were washed three times for 5 min in TBST, and were imaged and quantified using the FUSION SOLO 7S SYSTEM and the Fusion-Capt Advance 17.01 software (Vilber-Lourmat).

### Histochemical and spectrophotometric assay for surface localization of esterase activity

The qualitative esterase assay was modified from [50]. The assay medium contained 3.4 mM indoxyl acetate (Wako Chemicals) dissolved in 20 mM Tris-HCl, pH 8.0, containing 0.99 M NaCl, 44.6 mM CaCl_2_, and 17.5% gelatin. Hydrolysis of the substrate for esterase results in the accumulation of pigmented crystals of indigo blue at the site of hydrolysis. Conidia of *C*. *orbiculare* were placed on glass slides coated with a thin layer of assay medium; after a 2-h incubation in a humid box at 24°C under continuous light, conidia were examined. Cellular localization of esterase activity was determined by comparing unwashed spores and spores that had been washed 5 times for 1 min by vortex in 1 mL of a 0.01% Tween 20 solution in 20 mM Tris-HCl, pH 8.0. Autoclaved spores were included as a negative control of hydrolysis.

For collecting the extracellular matrix that surrounds spores, 100 mg of spores were repeatedly washed by vortexing for 1 min in 20 mM Tris-HCl, pH 8.0, containing 0.01% v/v Tween 20. The spore suspensions were then centrifuged, and collected supernatants were filtered through a 0.2-μm-pore filter. Esterase activity was determined by measuring the hydrolysis of *p*-nitrophenyl butyrate (Sigma) at 400 nm as described previously [50]. Assays were run at 37°C for 10 min.

### Detection of esterase activity by native polyacrylamide gel electrophoresis

Proteins washed from 220 mg of spores were freeze-dried, then dissolved in 500 μL of 50 mM Tris-HCl, pH 7.4, containing 150 mM NaCl, 1 mM EDTA, 1% v/v Triton X-100, 0.1 M PMSF, and a protease inhibitor cocktail tablet (Roche Diagnostics). Intracellular proteins were isolated from 220 mg of washed spores by grinding in liquid nitrogen. The tissue powder was dissolved in 500 μL of the same buffer as the extracellular proteins. The lysate was clarified by centrifugation (16,900 × *g*, 4°C, 20 min), and the supernatant was collected. Protein concentration was determined using the Bradford protein assay. Esterase from porcine liver (Sigma) was used as a positive control of hydrolysis.

Proteins were separated using a Novex Tris-Glycine gel (Thermo Fisher Scientific) and Tris-Glycine Native Sample Buffer (Thermo Fisher Scientific). Preparations containing 1.0, 0.1, or 0.05 μg proteins were loaded onto the gel. SeeBlue Pre-stained Protein Standard (Thermo Fisher Scientific) was used as the molecular weight marker. Electrophoresis was conducted at constant 125 V for 3 h at 4°C in 1× Tris-glycine native running buffer (Thermo Fisher Scientific). The gel was then washed twice for 10 min each in 100 mM Tris-HCI, pH 8.0. Esterase activity was detected using the indoxyl acetate assay as described [50].

### Microscopy

Bright-field microscopy was performed using a Nikon ECLIPSE E600 microscope equipped with a Keyence VB-7010 charge-coupled device (CCD) color camera system to acquire images with 20× and 40× water immersion lenses or a 100× oil immersion lens (Plan Fluor). Differential interference contrast (DIC) optics and a Zeiss Axio Imager M2 Upright microscope equipped with an AxioCam MRm digital camera were used to acquire images with a 100× oil immersion lens (Plan Apochromat) and Axiovision 4.8 software.

### Microarray analysis

For sampling of appressoria *in vitro*, 10 mL of a conidial suspension (5 × 10^5^ conidia/mL 0.1% w/v yeast extract solution) was placed on polystyrene petri dishes and incubated at 24°C in the dark for 1 h. The yeast extract solution was then removed and replaced with distilled water. After 3 h, the distilled water was removed, and cells were harvested using a scraper in 10 mL of RNAlater (Thermo Fisher Scientific) containing 1 mL of 0.01% (v/v) Tween 20. The conidia were collected by centrifugation at 15,000 rpm at 4°C for 10 min, then subjected to RNA isolation using an Agilent Plant RNA Isolation MiniKit (Agilent Technologies).

For sampling appressoria *in planta*, 10 μL of a conidial suspension (5 × 10^5^ conidia/mL distilled water) was spotted onto the abaxial surface of detached cucumber cotyledons and incubated in a humid box at 24°C in the dark. After 4 h, the lower epidermises of the cotyledons were peeled off and ground in liquid nitrogen. Then total RNA was prepared using the Agilent Plant RNA Isolation MiniKit.

Microarray analyses were performed using the *Colletotrichum orbiculare* (8 × 60k, 13352 independent probes, Design ID: 060762) Oligo Microarray, according to the Agilent 60-mer Oligo Microarray Processing Protocol (Agilent Technologies). The quality of total RNA was monitored with the Agilent 2200 Tapestation. Total RNA samples (200 ng) were used to prepare Cy3-labeled cRNA using a Low RNA Input Fluorescent Linear Amplification Kit (Agilent). Fluorescence-labeled cRNAs were purified using an RNeasy RNA Purification Kit (Qiagen). Three independent RNA samples were used to confirm the reproducibility of the microarray analyses. The images were analyzed using Feature Extraction Software (Ver. 10.7.3.1) and GeneSpring GX 12.1 software (Agilent). Normalization was performed as follows: (i) intensity-dependent Lowess normalization; (ii) data transformation, measurements less than 0.01 were set to 0.01; (iii) per-chip 75th-percentile normalization of each array; and (iv) per gene (data were normalized to the median value of 12 measurements). After normalization, statistically significant (t-test) gene sets were defined as those showing P values less than 0.05. In addition, the differentially expressed genes (>2-fold change) were identified. Thus, a combination of statistical analysis and FC method was used.　The differentially regulated genes (FC > 2 and *P* < 0.05) were selected and used for further analysis. The raw and processed data were deposited in the Gene Expression Omnibus (GEO) database (access ID: GSE 7556).

### Accession numbers

Sequence data from this article can be found in the GenBank databases under the following accession numbers: CoPag1 (ENH85423), CoCbk1 (ENH84264), CoHym1 (LC147073), CoKel2 (AB259753), CoCac1 (AB127957), CoCmk1 (AF174649), CoMekk1 (AB546841), *S*. *cerevisiae* Tao3 (NP_012137), Cbk1 (NP_014238) and Hym1 (NP_012732). Accession numbers of Pag1 homologs of other fungi and eukaryotes are as follows: *C*. *higginsianum* (CCF34414); *C*. *graminicola* (EFQ30591); *Neurospora crassa* (XP_964442); *Magnaporthe oryzae* (XP_003715305); *Aspergillus fumigatus* (XP_750987); *A*. *nidulans* (CBF89162); *Candida albicans* Tao3 (XP_721647); *Schizosaccharomyces pombe* Mor2 (NP_596172); *Cryptococcus neoformans* Tao3 (ADY38376); *Ustilago maydis* Tao3 (XP_011386655); *Caenorhabditis elegans* SAX-2 (NP_741131); *Drosophila melanogaster* Fry (AAG41424); *Homo sapiens* FRY (NP_075463).

## Supporting information

S1 Fig Characterization of pathogenesis deficient mutants (PDM) of *C*. *orbiculare*. (A) Appressorium development of PDMs on glass slides after 24 h at 24°C. Conidial suspensions of strains were prepared with distilled water. WT, wild type strain 104-T; *kel2Δ*, *cokel2* mutant strain; PDM-2, 3, 4, 5, 6, 7, six insertional mutant strains in *cokel2Δ* background; PDMs/*PAG1*, *CoPAG1* complemented strains in PDMs. Scale bar, 10 μm. Co, conidium; Ap, appressorium. (B) Mean percentage (±SE) of normal appressorium formation of PDMs on glass slide at 24 h after inoculation. At least 300 conidia on a glass slide were observed in each of three independent experiments. Values are means of three replications. Bars with different letters indicate significant differences (Tukey’s test; *P* < 0.01). (C) Development of infection structures of PDMs on lower surface of detached cucumber cotyledons at 3 d after inoculation. Penetration hyphae were stained with lactophenol aniline blue. WT, wild type strain 104-T; *kel2Δ*, *cokel2* mutant strain; PDM-2, 3, 4, 5, 6, 7, six insertional mutant strains in *cokel2Δ* background; PDMs/*PAG1*, *CoPAG1* complemented strains in PDMs; PDMs/Cbk1-CA, CoCbk1-CA strains in PDMs. Ap, appressorium; Ph, penetration hypha. Scale bar, 10 μm. (D) Percentage of normal appressoria and penetration hyphae formed on lower surface of detached cucumber cotyledons. At least 300 appressoria on three cotyledons were observed at each experiment, and three independent experiments were performed. Values are means of three replications; error bars represent ±SE. Bars with different letters indicate significant differences (Tukey’s test; *P* < 0.05). (E) Pathogenicity assay of PDMs on intact cucumber cotyledons after 6 d at 24°C. Conidial suspensions of indicated strains were prepared in distilled water and dropped onto detached cucumber cotyledons. (F) Hypothetical relation between mutated genes of PDMs and plant-derived signal transduction for appressorium morphogenesis. Whereas introduction of CoCbk1-CA to PDM-2, 3, 4, 7 restored normal appressorium formation, phenotypes of Cbk1-CA strains in PDM-5, 6 were not affected. Thus, it could be hypothesized that the disrupted genes of PDM-2, 3, 7 were upstream and PDM-5, 6 were downstream of CoCbk1 respectively.
(PDF)

S2 Fig *C*. *orbiculare PAG1* is a functional ortholog of *S*. *cerevisiae TAO3*. (A) Schematic representation of *C*. *orbiculare* Pag1 protein. *CoPAG1* putatively encodes a 2419-amino-acid protein with MOR2-PAG1_N, MOR2-PAG1_mid and MOR2-PAG1_C domains recognized in the Pfam protein families database. (B) Phylogenetic tree of *C*. *orbiculare* Pag1 with Tao3 homologs of other fungi and eukaryotes. *Colletotrichum orbiculare* Pag1 (ENH85423), *Colletotrichum higginsianum* (CCF34414), *Colletotrichum graminicola* (EFQ30591), *Neurospora crassa* (XP964442), *Magnaporthe oryzae* (XP003715305), *Aspergillus fumigatus* (XP750987), *Aspergillus nidulans* (CBF89162), *Saccharomyces cerevisiae* Tao3 (NP012137), *Candida albicans* Tao3 (XP721647), *Schizosaccharomyces pombe* Mor2 (NP596172), *Cryptococcus neoformans* Tao3 (ADY38376), *Ustilago maydis* Tao3 (XP011386655), *Caenorhabditis elegans* SAX-2 (NP741131), *Drosophila melanogaster* Fry (AAG41424) and *Homo sapiens* FRY (NP075463). Bootstrap values are shown; scale bar denotes evolutionary distances. Red, filamentous ascomycetes; blue, ascomycetous yeasts; green, basidiomycetes; black, other eukaryotes. (C) Complementation of *S*. *cerevisiae tao3* by *C*. *orbiculare PAG1* cDNA. Strains containing *GAL1*_*pro*_:*TAO3* or *GAL1*_*pro*_:*CoPAG1* were induced by adding galactose. WT, *S*. *cerevisiae* wild type strain W303-1A; *tao3Δ*, *tao3*-deficient *S*. *cerevisiae* strain FLY1004, *tao3Δ/TAO3*, *tao3Δ* transformants expressing the *TAO3* of *S*. *cerevisiae*, *tao3Δ/CoPAG1*, *tao3Δ* transformants expressing the *PAG1* cDNA of *C*. *orbiculare*. Scale bar, 10 μm. (D) Mean axial ratio (length/width) (±SE) of yeast cells. Means are from three independent experiments each with at least 100 cells. Values with different letters differed significantly from each other (Tukey’s test; *P* < 0.01).
(PDF)

S3 Fig Southern blot analysis of targeted gene deletion mutants. (A) Gene deletion strategy of *PAG1* in *C*. *orbiculare* by homologous recombination. Bars indicate probes used for DNA gel blots. *HPH*, hygromycin B phosphotransferase gene. (B) DNA gel blot analysis of *CoPAG1* transformants. Genomic DNA was digested with HindIII and EcoRI and probed with an upstream fragment of *CoPAG1*. D1-3, *copag1Δ*; E1, *copag1Δ* ectopic transformants; D4-6, *copag1Δ cokel2Δ*; E2, *copag1Δ cokel2Δ* ectopic transformants; WT, wild type strain 104-T. (C) Gene deletion strategy of *HYM1* in *C*. *orbiculare* by homologous recombination. Bars indicate probes used for DNA gel blots. (D) DNA gel blot analysis of *CoHYM1* transformants. Genomic DNA was digested with EcoRV and probed with a downstream fragment of *CoHYM1*. D1-3, *cohym1Δ*; E1, *cohym1Δ* ectopic transformants; WT, wild type strain 104-T.
(PDF)

S4 Fig Appressorium morphogenesis and pathogenicity of the *copag1* deletion mutants on *N*. *benthamiana*. (A) Development of infection structures of wild type strain 104-T (WT) and *cokel2* and *copag1* mutant strains on upper surface of *N*. *benthamiana* leaves at 3 d after inoculation. Penetration hyphae were stained with lactophenol trypan blue. Co, conidium; Ap, appressorium; Ph, penetration hypha. Scale bar, 10 μm. (B) Percentage of normal appressoria and penetration hyphae formed on upper surface of *N*. *benthamiana* leaves. At least 300 appressoria on a leaf were observed at each experiment, and three independent experiments were performed. Values are the means of three replications; error bars represent ±SE. Bars with different letters indicate significant differences (Tukey’s test; *P* < 0.01). (C) Pathogenicity assays on *N*. *benthamiana*. Conidial suspensions of the respective strains were dropped onto upper surface of leaves of 4-week-old plants and incubated at 24°C for 4 d.
(PDF)

S5 Fig Amino acid sequence alignment of *C*. *orbiculare* (Co) Cbk1 and *S*. *cerevisiae* (Sc) Cbk1. The alignment was generated using Clustal W. Numbers on the right indicate amino acid residue positions. Shading of residues represents 100% amino acid conservation. Gaps introduced for alignments are indicated by a hyphen. The catalytic domain of fungal nuclear Dbf2-related kinase-like protein and the hydrophobic motif (HM) are indicated.
(PDF)

S6 Fig MOR component CoHym1 functions *via* activation of CoCbk1 in *C*. *orbiculare*. (A) The amino acid sequence alignment of *C*. *orbiculare* (Co) Hym1 and *S*. *cerevisiae* (Sc) Hym1 was generated using Clustal W. Numbers on the right indicate amino acid residue positions. Shading of residues represents 100% amino acid conservation. Gaps introduced for alignments are indicated by a hyphen. Mo25 domain, which is conserved in Hym1 homologous proteins, is indicated. (B) Appressorium development of *cohym1Δ* and *cohym1Δ*/CoCbk1-CA on petri dishes after 24 h at 24°C. Conidial suspensions of strains were prepared with distilled water. Co, conidium; Ap, appressorium. Scale bar, 10 μm. (C) Mean percentage (±SE) of normal appressorium formation of *cohym1Δ* and *cohym1Δ*/CoCbk1-CA on petri dishes at 24 h after inoculation. At least 300 appressoria on a petri dish were observed in each of three independent experiments. Values are means of three replications. Bars with different letters indicate significant differences (Tukey’s test; *P* < 0.01).
(PDF)

S7 Fig CoCbk1 interacts with CoPag1 and CoHym1 in yeast two- hybrid assays. CoPag1, CoHym1 and CoKel2 were expressed in fusion with Gal4 DNA-binding domain (BD) protein and their interaction with CoCbk1 fused with Gal4 activation domain (AD) were tested by yeast two-hybrid assay. Interaction was assessed from yeast growth on SD media lacking–Trp–Leu (DDO),–Trp–Leu–Ade–His (QDO), and–Trp–Leu + X-α-Gal + AbA (DDO/X/A). Plasmids expressing the indicated proteins either as prey or as bait alone were used as negative controls and pGBKT7-53 (murine p53) and pGADT7-recT (SV40 large T antigen) fusion proteins as positive control. +, Interaction;–, no interaction.
(PDF)

S8 Fig Constitutive activation of CoCbk1 had no effect on the cAMP and MAP kinase signaling cascades in *C*. *orbiculare*. (A) Conidial germination and appressorium formation of CoCbk1-CA strains on glass slides and lower surface of detached cucumber cotyledons after 24 h at 24°C. Conidial suspensions of strains were prepared with distilled water. Scale bar, 10 μm. Co, conidium; Ap, appressorium. (B) Mean percentage (±SE) of conidial germination and appressorium formation of CoCbk1-CA strains on glass slide at 24 h after inoculation. At least 300 conidia on a glass slide were observed in each of three independent experiments. Values are means of three replications. Bars with different letters indicate significant differences (Tukey’s test; *P* < 0.01). (C) Pathogenicity assay of CoCbk1-CA strains on intact cucumber cotyledons after 7 d at 24°C. Conidial suspensions of indicated strains were prepared in distilled water and dropped onto detached cucumber cotyledons.
(PDF)

S1 Table Phenotypes of the mutants used in this study. (PDF)

S2 Table *Colletotrichum orbiculare* strains used in this study. (PDF)

S3 Table *Saccharomyces cerevisiae* strains used in this study. (PDF)

S4 Table PCR primers used in this study. (PDF)

S1 Data Set Differentially regulated genes in strain CoCbk1-CA in the *copag1Δ* mutant background compared with the wild type during appressorium development *in vitro*. (XLSX)
