# Supplementary material for: The morphogenesis-related NDR kinase pathway of Colletotrichum orbiculare is required for translating plant surface signals into infection-related morphogenesis and pathogenesis
Source: PLoS Pathog. 2017 Feb 1;13(2):e1006189. doi: 10.1371/journal.ppat.1006189 (PMC5305266; doi:10.1371/journal.ppat.1006189)
Supplement: S1 Fig — (A) Appressorium development of PDMs on glass slides after 24 h at 24°C. Conidial suspensions of strains were prepared with distilled water. WT, wild type strain 104-T; kel2Δ, cokel2 mutant strain; PDM-2, 3, 4, 5, 6, 7, six insertional mutant strains in cokel2Δ background; PDMs/PAG1, CoPAG1 complemented strains in PDMs. Scale bar, 10 μm. Co, conidium; Ap, appressorium. (B) Mean percentage (±SE) of normal appressorium formation of PDMs on glass slide at 24 h after inoculation. At least 300 conidia on a glass slide were observed in each of three independent experiments. Values are means of three replications. Bars with different letters indicate significant differences (Tukey’s test; P < 0.01). (C) Development of infection structures of PDMs on lower surface of detached cucumber cotyledons at 3 d after inoculation. Penetration hyphae were stained with lactophenol aniline blue. WT, wild type strain 104-T; kel2Δ, cokel2 mutant strain; PDM-2, 3, 4, 5, 6, 7, six insertional mutant strains in cokel2Δ background; PDMs/PAG1, CoPAG1 complemented strains in PDMs; PDMs/Cbk1-CA, CoCbk1-CA strains in PDMs. Ap, appressorium; Ph, penetration hypha. Scale bar, 10 μm. (D) Percentage of normal appressoria and penetration hyphae formed on lower surface of detached cucumber cotyledons. At least 300 appressoria on three cotyledons were observed at each experiment, and three independent experiments were performed. Values are means of three replications; error bars represent ±SE. Bars with different letters indicate significant differences (Tukey’s test; P < 0.05). (E) Pathogenicity assay of PDMs on intact cucumber cotyledons after 6 d at 24°C. Conidial suspensions of indicated strains were prepared in distilled water and dropped onto detached cucumber cotyledons. (F) Hypothetical relation between mutated genes of PDMs and plant-derived signal transduction for appressorium morphogenesis. Whereas introduction of CoCbk1-CA to PDM-2, 3, 4, 7 restored normal appressorium formation, phenotypes [file ppat.1006189.s001.pdf]

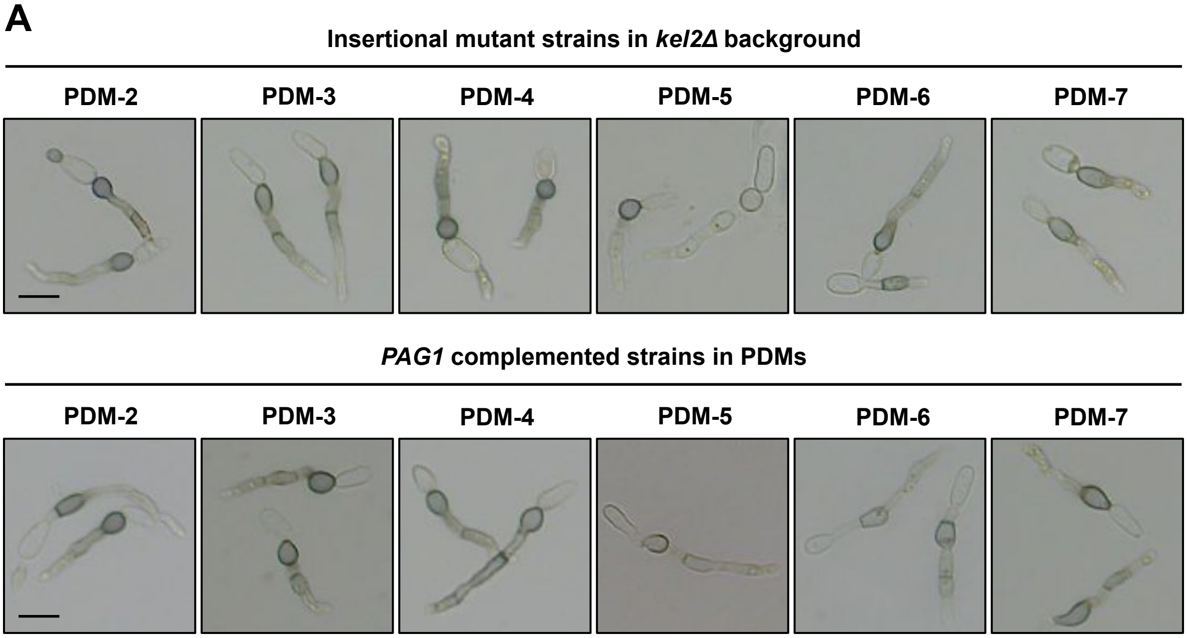

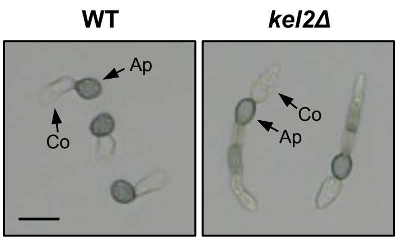

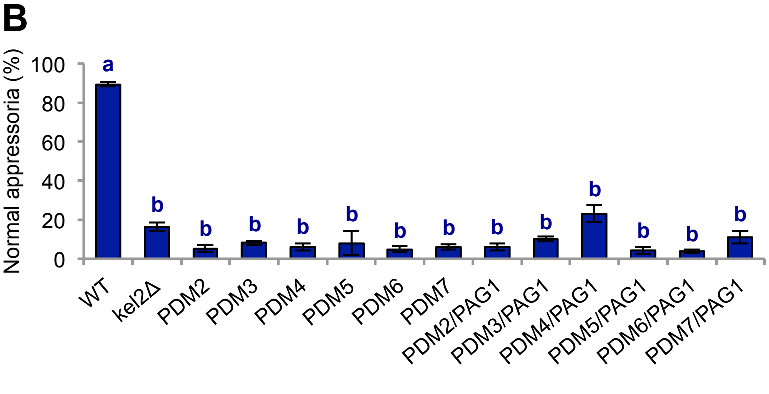

Continued on next page

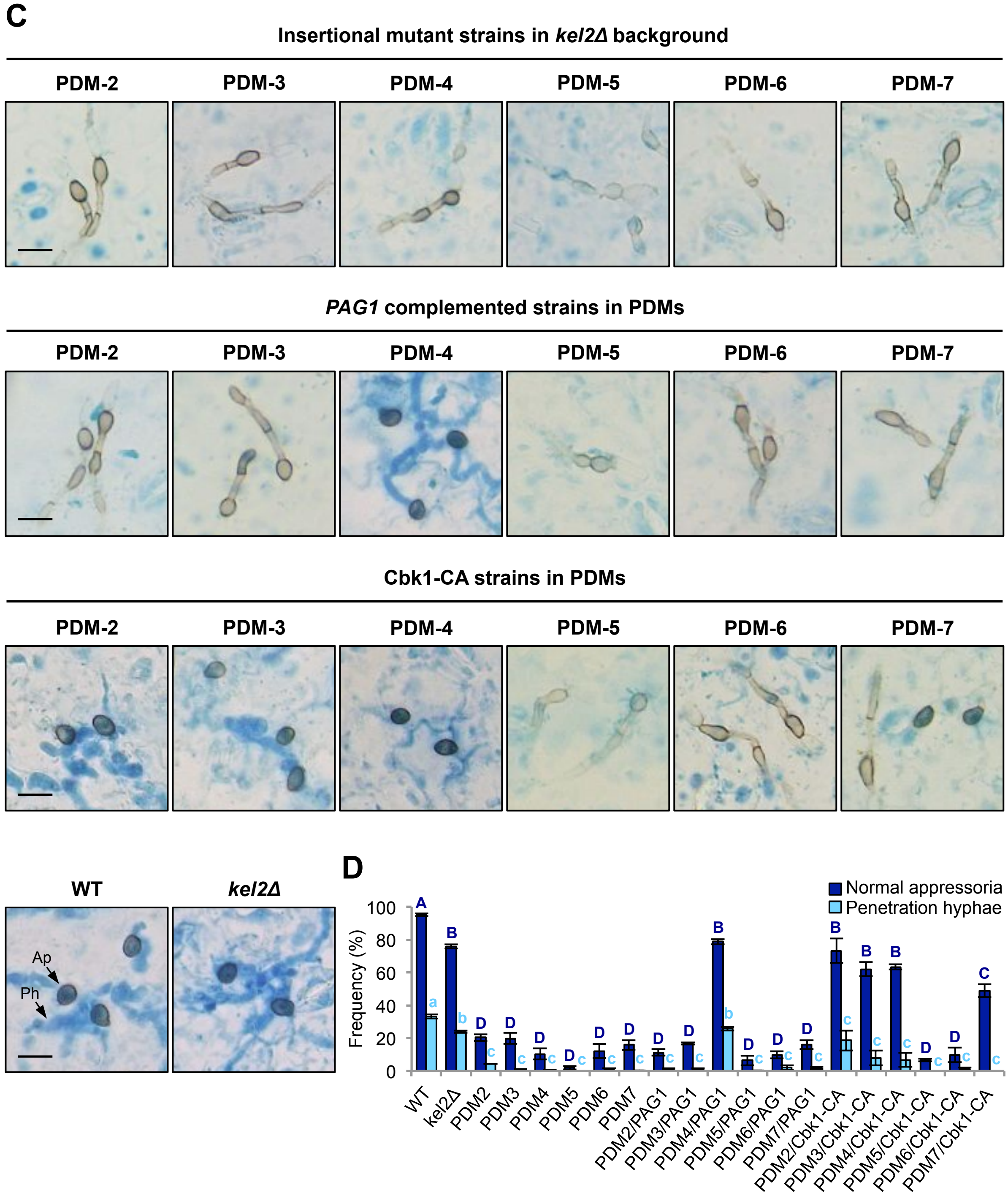

Continued on next page

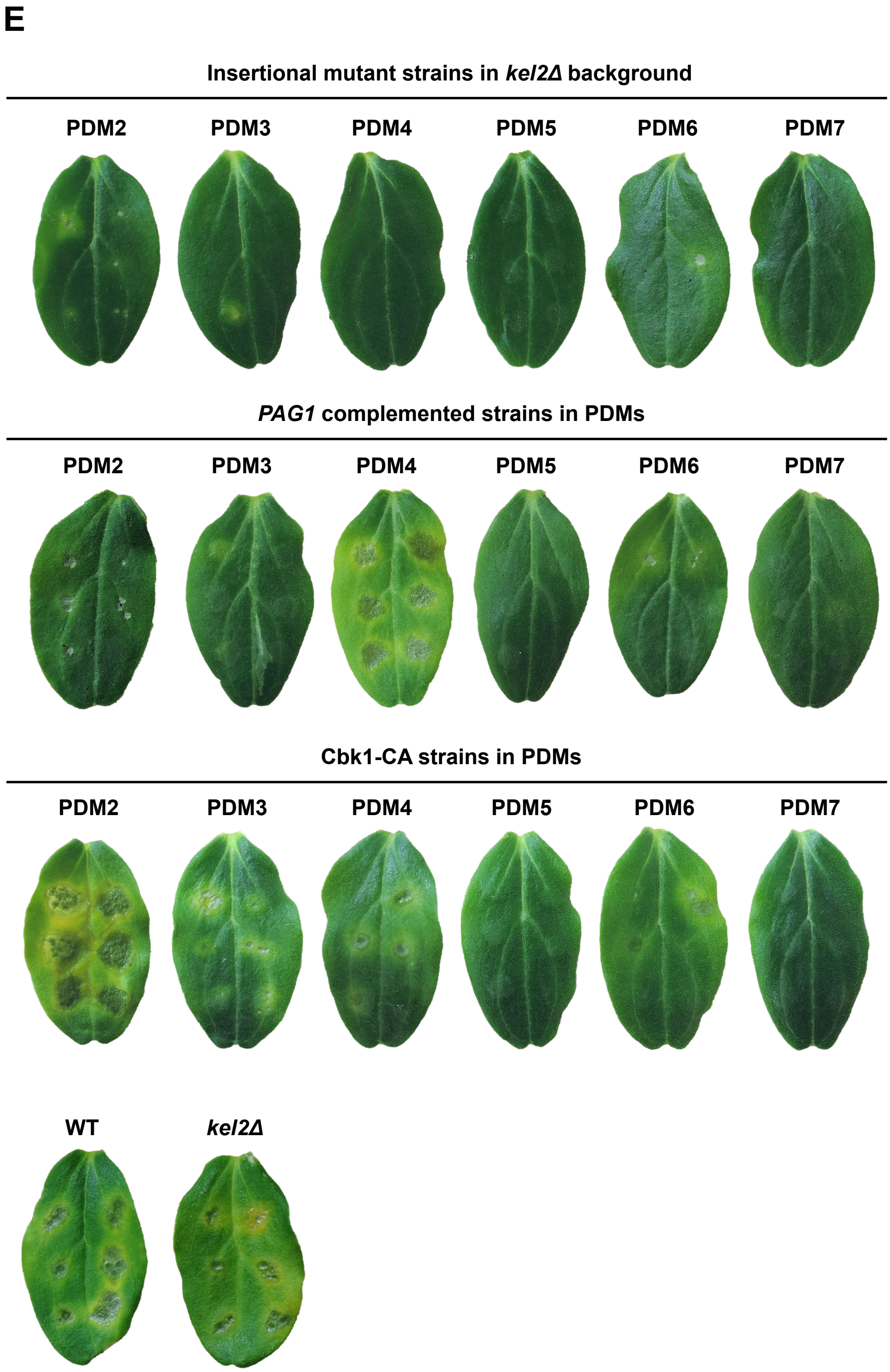

Continued on next page

F

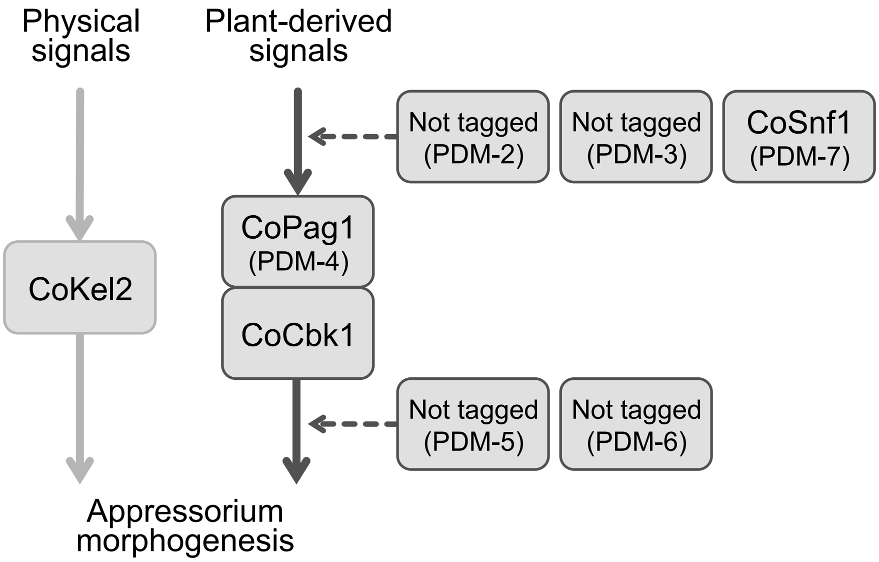
Physical signals
Plant-derived signals
Not tagged (PDM-2)
Not tagged (PDM-3)
CoSnf1 (PDM-7)
CoPag1 (PDM-4)
CoKel2
CoCbk1
Not tagged (PDM-5)
Not tagged (PDM-6)
Appressorium morphogenesis
