# Supplementary figures and images for: The morphogenesis-related NDR kinase pathway of Colletotrichum orbiculare is required for translating plant surface signals into infection-related morphogenesis and pathogenesis
Source: PLoS Pathog. 2017 Feb 1;13(2):e1006189. doi: 10.1371/journal.ppat.1006189 (PMC5305266; doi:10.1371/journal.ppat.1006189)

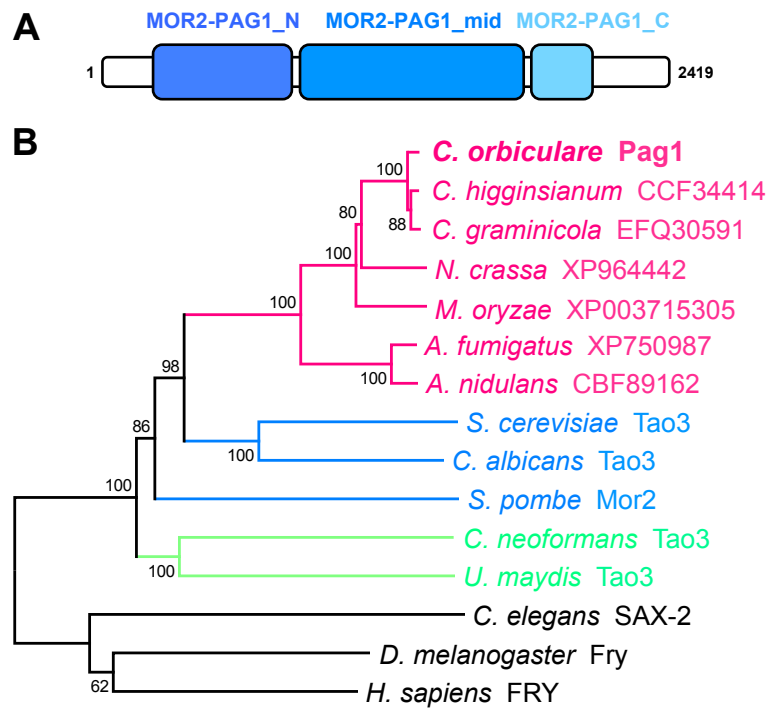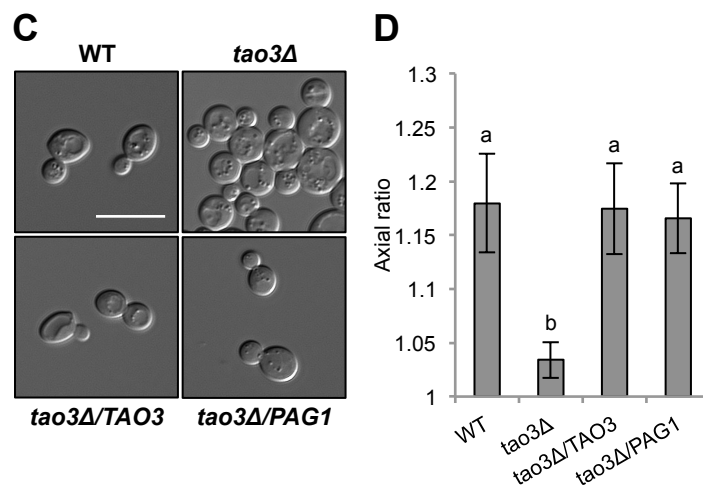

Supplement: S2 Fig — (A) Schematic representation of C. orbiculare Pag1 protein. CoPAG1 putatively encodes a 2419-amino-acid protein with MOR2-PAG1_N, MOR2-PAG1_mid and MOR2-PAG1_C domains recognized in the Pfam protein families database. (B) Phylogenetic tree of C. orbiculare Pag1 with Tao3 homologs of other fungi and eukaryotes. Colletotrichum orbiculare Pag1 (ENH85423), Colletotrichum higginsianum (CCF34414), Colletotrichum graminicola (EFQ30591), Neurospora crassa (XP964442), Magnaporthe oryzae (XP003715305), Aspergillus fumigatus (XP750987), Aspergillus nidulans (CBF89162), Saccharomyces cerevisiae Tao3 (NP012137), Candida albicans Tao3 (XP721647), Schizosaccharomyces pombe Mor2 (NP596172), Cryptococcus neoformans Tao3 (ADY38376), Ustilago maydis Tao3 (XP011386655), Caenorhabditis elegans SAX-2 (NP741131), Drosophila melanogaster Fry (AAG41424) and Homo sapiens FRY (NP075463). Bootstrap values are shown; scale bar denotes evolutionary distances. Red, filamentous ascomycetes; blue, ascomycetous yeasts; green, basidiomycetes; black, other eukaryotes. (C) Complementation of S. cerevisiae tao3 by C. orbiculare PAG1 cDNA. Strains containing GAL1pro:TAO3 or GAL1pro:CoPAG1 were induced by adding galactose. WT, S. cerevisiae wild type strain W303-1A; tao3Δ, tao3-deficient S. cerevisiae strain FLY1004, tao3Δ/TAO3, tao3Δ transformants expressing the TAO3 of S. cerevisiae, tao3Δ/CoPAG1, tao3Δ transformants expressing the PAG1 cDNA of C. orbiculare. Scale bar, 10 μm. (D) Mean axial ratio (length/width) (±SE) of yeast cells. Means are from three independent experiments each with at least 100 cells. Values with different letters differed significantly from each other (Tukey’s test; P < 0.01). (PDF) [file ppat.1006189.s002.pdf]

**A**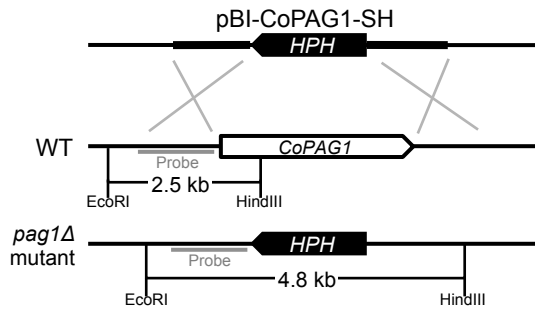**B**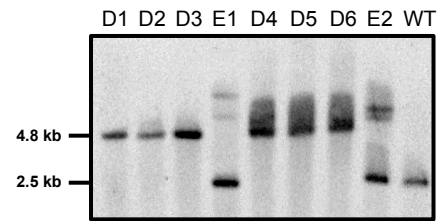**C**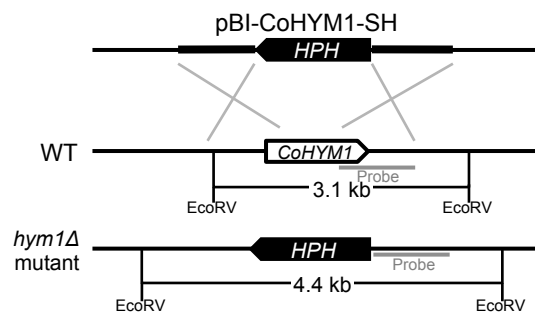**D**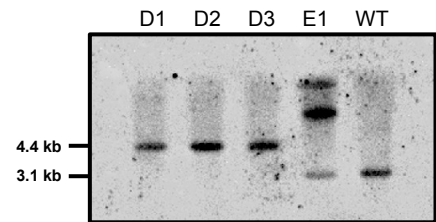

Supplement: S3 Fig — (A) Gene deletion strategy of PAG1 in C. orbiculare by homologous recombination. Bars indicate probes used for DNA gel blots. HPH, hygromycin B phosphotransferase gene. (B) DNA gel blot analysis of CoPAG1 transformants. Genomic DNA was digested with HindIII and EcoRI and probed with an upstream fragment of CoPAG1. D1-3, copag1Δ; E1, copag1Δ ectopic transformants; D4-6, copag1Δ cokel2Δ; E2, copag1Δ cokel2Δ ectopic transformants; WT, wild type strain 104-T. (C) Gene deletion strategy of HYM1 in C. orbiculare by homologous recombination. Bars indicate probes used for DNA gel blots. (D) DNA gel blot analysis of CoHYM1 transformants. Genomic DNA was digested with EcoRV and probed with a downstream fragment of CoHYM1. D1-3, cohym1Δ; E1, cohym1Δ ectopic transformants; WT, wild type strain 104-T. (PDF) [file ppat.1006189.s003.pdf]

**A**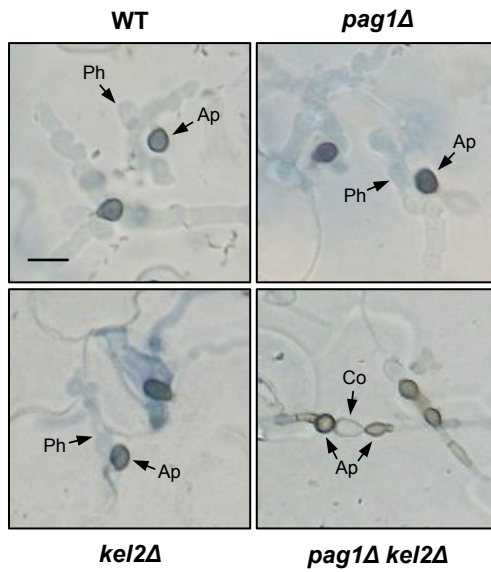**B**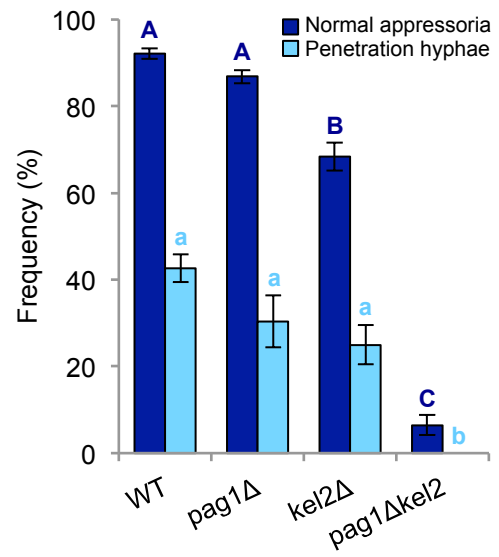**C**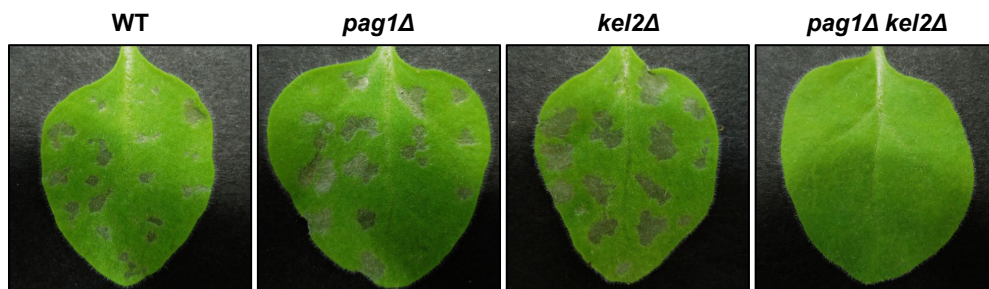

Supplement: S4 Fig — (A) Development of infection structures of wild type strain 104-T (WT) and cokel2 and copag1 mutant strains on upper surface of N. benthamiana leaves at 3 d after inoculation. Penetration hyphae were stained with lactophenol trypan blue. Co, conidium; Ap, appressorium; Ph, penetration hypha. Scale bar, 10 μm. (B) Percentage of normal appressoria and penetration hyphae formed on upper surface of N. benthamiana leaves. At least 300 appressoria on a leaf were observed at each experiment, and three independent experiments were performed. Values are the means of three replications; error bars represent ±SE. Bars with different letters indicate significant differences (Tukey’s test; P < 0.01). (C) Pathogenicity assays on N. benthamiana. Conidial suspensions of the respective strains were dropped onto upper surface of leaves of 4-week-old plants and incubated at 24°C for 4 d. (PDF) [file ppat.1006189.s004.pdf]

**A**

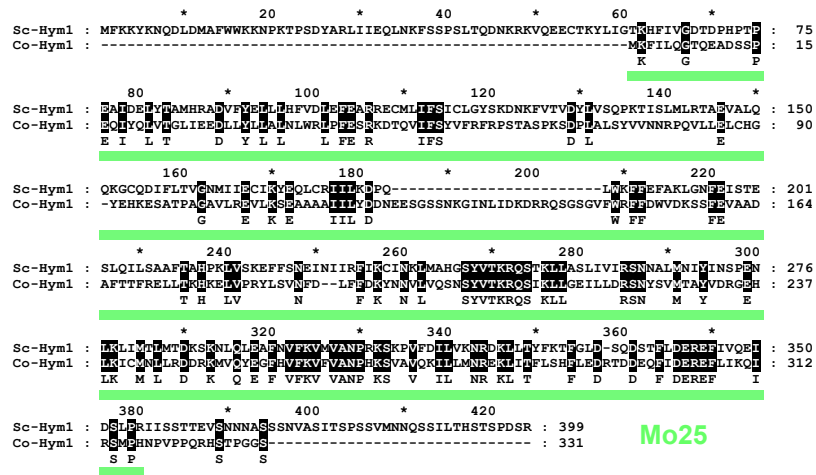

**B**

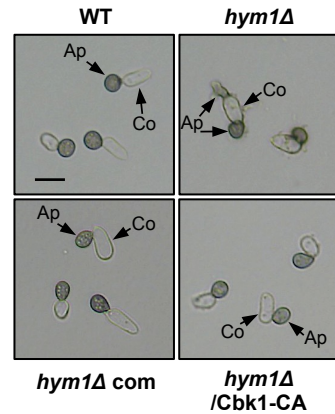

**C**

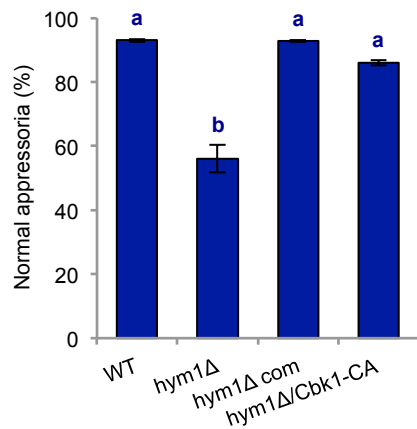

Supplement: S6 Fig — (A) The amino acid sequence alignment of C. orbiculare (Co) Hym1 and S. cerevisiae (Sc) Hym1 was generated using Clustal W. Numbers on the right indicate amino acid residue positions. Shading of residues represents 100% amino acid conservation. Gaps introduced for alignments are indicated by a hyphen. Mo25 domain, which is conserved in Hym1 homologous proteins, is indicated. (B) Appressorium development of cohym1Δ and cohym1Δ/CoCbk1-CA on petri dishes after 24 h at 24°C. Conidial suspensions of strains were prepared with distilled water. Co, conidium; Ap, appressorium. Scale bar, 10 μm. (C) Mean percentage (±SE) of normal appressorium formation of cohym1Δ and cohym1Δ/CoCbk1-CA on petri dishes at 24 h after inoculation. At least 300 appressoria on a petri dish were observed in each of three independent experiments. Values are means of three replications. Bars with different letters indicate significant differences (Tukey’s test; P < 0.01). (PDF) [file ppat.1006189.s006.pdf]

**A**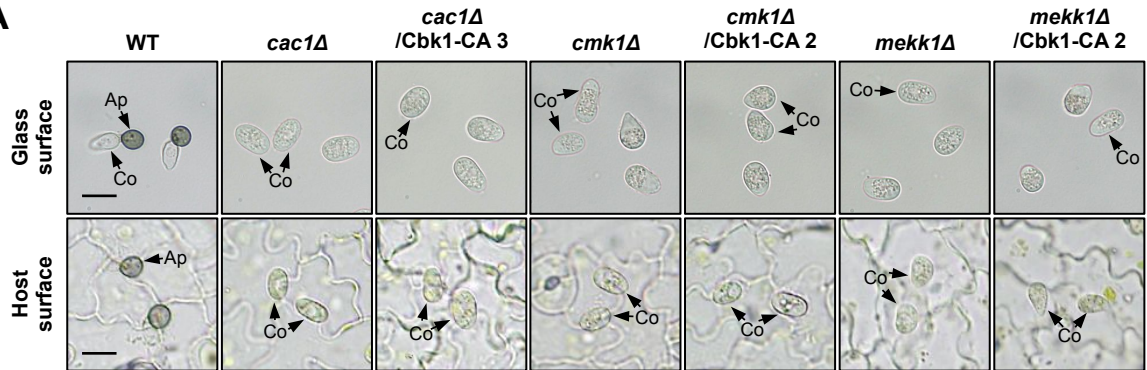**B**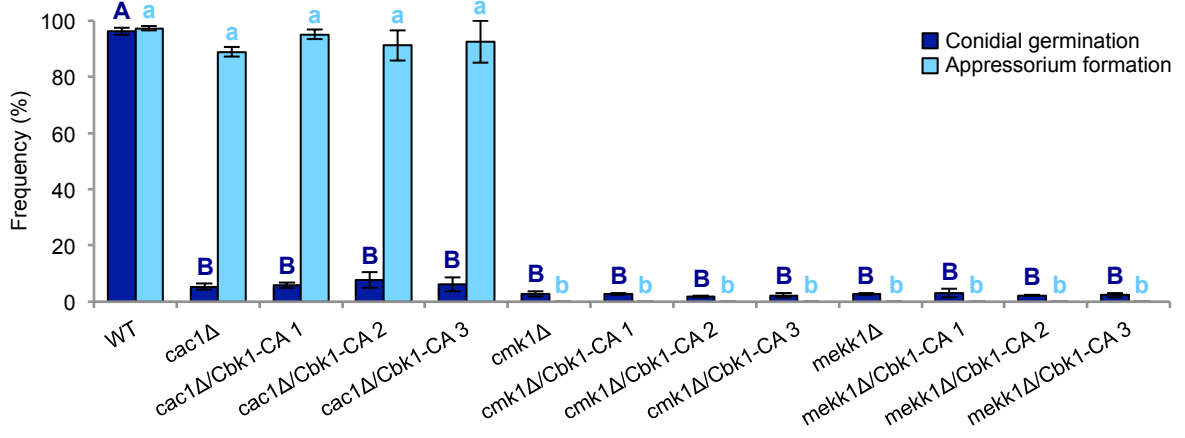**C**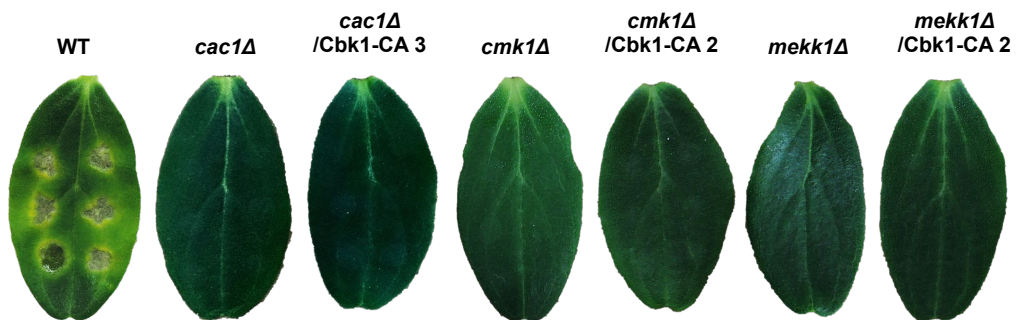

Supplement: S8 Fig — (A) Conidial germination and appressorium formation of CoCbk1-CA strains on glass slides and lower surface of detached cucumber cotyledons after 24 h at 24°C. Conidial suspensions of strains were prepared with distilled water. Scale bar, 10 μm. Co, conidium; Ap, appressorium. (B) Mean percentage (±SE) of conidial germination and appressorium formation of CoCbk1-CA strains on glass slide at 24 h after inoculation. At least 300 conidia on a glass slide were observed in each of three independent experiments. Values are means of three replications. Bars with different letters indicate significant differences (Tukey’s test; P < 0.01). (C) Pathogenicity assay of CoCbk1-CA strains on intact cucumber cotyledons after 7 d at 24°C. Conidial suspensions of indicated strains were prepared in distilled water and dropped onto detached cucumber cotyledons. (PDF) [file ppat.1006189.s008.pdf]
