# Supplementary material for: The morphogenesis-related NDR kinase pathway of Colletotrichum orbiculare is required for translating plant surface signals into infection-related morphogenesis and pathogenesis
Source: PLoS Pathog. 2017 Feb 1;13(2):e1006189. doi: 10.1371/journal.ppat.1006189 (PMC5305266; doi:10.1371/journal.ppat.1006189)
Supplement: S5 Fig — The alignment was generated using Clustal W. Numbers on the right indicate amino acid residue positions. Shading of residues represents 100% amino acid conservation. Gaps introduced for alignments are indicated by a hyphen. The catalytic domain of fungal nuclear Dbf2-related kinase-like protein and the hydrophobic motif (HM) are indicated. (PDF) [file ppat.1006189.s005.pdf]

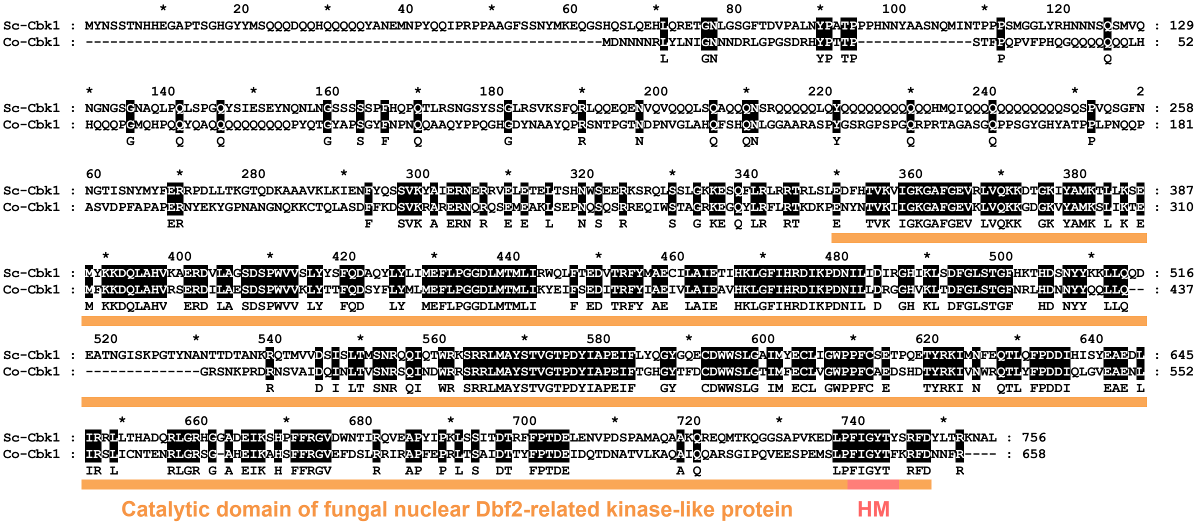
Sc-Cbk1
Co-Cbk1
Catalytic domain of fungal nuclear Dbf2-related kinase-like protein
HM
