# Supplementary material for: The morphogenesis-related NDR kinase pathway of Colletotrichum orbiculare is required for translating plant surface signals into infection-related morphogenesis and pathogenesis
Source: PLoS Pathog. 2017 Feb 1;13(2):e1006189. doi: 10.1371/journal.ppat.1006189 (PMC5305266; doi:10.1371/journal.ppat.1006189)
Supplement: S7 Fig — CoPag1, CoHym1 and CoKel2 were expressed in fusion with Gal4 DNA-binding domain (BD) protein and their interaction with CoCbk1 fused with Gal4 activation domain (AD) were tested by yeast two-hybrid assay. Interaction was assessed from yeast growth on SD media lacking–Trp–Leu (DDO),–Trp–Leu–Ade–His (QDO), and–Trp–Leu + X-α-Gal + AbA (DDO/X/A). Plasmids expressing the indicated proteins either as prey or as bait alone were used as negative controls and pGBKT7-53 (murine p53) and pGADT7-recT (SV40 large T antigen) fusion proteins as positive control. +, Interaction;–, no interaction. (PDF) [file ppat.1006189.s007.pdf]

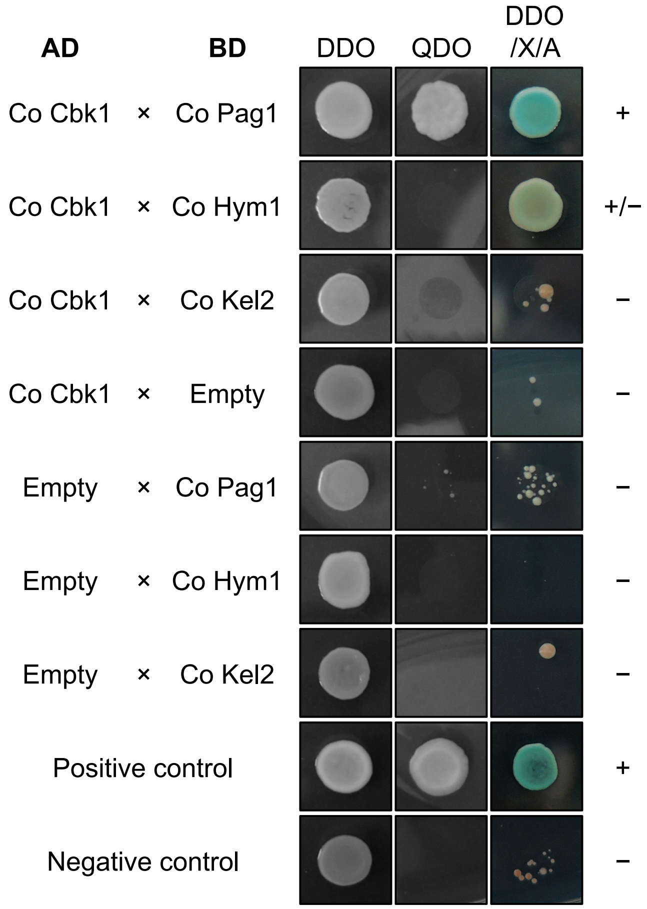

AD
BD
DDO
QDO
DDO /X/A
Co Cbk1 × Co Pag1 +
Co Cbk1 × Co Hym1 +/−
Co Cbk1 × Co Kel2 −
Co Cbk1 × Empty −
Empty × Co Pag1 −
Empty × Co Hym1 −
Empty × Co Kel2 −
Positive control +
Negative control −
