# Supplementary material for: The morphogenesis-related NDR kinase pathway of Colletotrichum orbiculare is required for translating plant surface signals into infection-related morphogenesis and pathogenesis
Source: PLoS Pathog. 2017 Feb 1;13(2):e1006189. doi: 10.1371/journal.ppat.1006189 (PMC5305266; doi:10.1371/journal.ppat.1006189)
Supplement: S1 Table — (PDF) [file ppat.1006189.s009.pdf]

S1 Table. Phenotypes of the mutants used in this study.

Strain	*in vitro*	*in planta*	+ *n*-Octadecanal *in vitro*	Pathogenesis
Wild type	N	N	N	+++
cokel2Δ	A	N	N	+++
copag1Δ	N	N	N	++
copag1Δ cokel2Δ	A	A	A	-
CoCbk1-AS (+ 1NA-PP1)	A	A	ND	-
Wild type/CoCbk1-CA	N	N	ND	++
copag1Δ cokel2Δ/CoCbk1-CA	N	N	ND	+

N: Normal appressorium, A: Abnormal appressorium, ND: No Data
