# Supplementary material for: The morphogenesis-related NDR kinase pathway of Colletotrichum orbiculare is required for translating plant surface signals into infection-related morphogenesis and pathogenesis
Source: PLoS Pathog. 2017 Feb 1;13(2):e1006189. doi: 10.1371/journal.ppat.1006189 (PMC5305266; doi:10.1371/journal.ppat.1006189)
Supplement: S2 Table — (PDF) [file ppat.1006189.s010.pdf]

S2 Table. ***Colletotrichum orbiculare*** **strains used in this study.**

Strain	Genotype description	Reference
104-T (MAFF240422)	Wild type	Ishida and Akai, 1969
kel2Δ	Wild type/*kel2Δ*	Sakaguchi et al., 2008
PDM-2	Insertional mutant in *kel2Δ*	This study
PDM-3	Insertional mutant in *kel2Δ*	This study
PDM-4	Insertional mutant in *kel2Δ*	This study
PDM-5	Insertional mutant in *kel2Δ*	This study
PDM-6	Insertional mutant in *kel2Δ*	This study
PDM-7	Insertional mutant in *kel2Δ*	This study
PDM-2/*PAG1*	*PAG1* complemented transformant in PDM-2	This study
PDM-3/*PAG1*	*PAG1* complemented transformant in PDM-3	This study
PDM-4/*PAG1*	*PAG1* complemented transformant in PDM-4	This study
PDM-5/*PAG1*	*PAG1* complemented transformant in PDM-5	This study
PDM-6/*PAG1*	*PAG1* complemented transformant in PDM-6	This study
PDM-7/*PAG1*	*PAG1* complemented transformant in PDM-7	This study
PDM-2/Cbk1-CA	Cbk1_T649E in PDM-2	This study
PDM-3/Cbk1-CA	Cbk1_T649E in PDM-3	This study
PDM-4/Cbk1-CA	Cbk1_T649E in PDM-4	This study
PDM-5/Cbk1-CA	Cbk1_T649E in PDM-5	This study
PDM-6/Cbk1-CA	Cbk1_T649E in PDM-6	This study
PDM-7/Cbk1-CA	Cbk1_T649E in PDM-7	This study
*pag1Δ*_1	Wild type/*pag1Δ*	This study
*pag1Δ*_2	Wild type/*pag1Δ*	This study
*pag1Δ*_3	Wild type/*pag1Δ*	This study
*pag1Δkel2Δ*_1	Wild type/*pag1Δkel2Δ*	This study
*pag1Δkel2Δ*_2	Wild type/*pag1Δkel2Δ*	This study
*pag1Δkel2Δ*_3	Wild type/*pag1Δkel2Δ*	This study
*pag1Δ*_ect	Ectopic transformant for *PAG1* deletion	This study
*pag1Δkel2Δ*_ect	Ectopic transformant for *PAG1 KEL2* deletion	This study
Cbk1-AS1	Wild type/Cbk1_M352A	This study
Cbk1-AS3	Wild type/Cbk1_M352A	This study
WT/Cbk1-CA	Wild type/Cbk1_T649E	This study
pag1Δ/Cbk1-CA	Wild type/*pag1Δ*/Cbk1_T649E	This study
pag1Δkel2Δ/Cbk1-CA	Wild type/*pag1Δkel2Δ*/Cbk1_T649E	This study
*hym1Δ*_1	Wild type/*hym1Δ*	This study
*hym1Δ*_2	Wild type/*hym1Δ*	This study
*hym1Δ*_3	Wild type/*hym1Δ*	This study
*hym1Δ*_ect	Ectopic transformant for *HYM1* deletion	This study
*hym1Δ*_com	Wild type/*hym1Δ*/*HYM1*	This study
hym1Δ/Cbk1-CA	Wild type/*hym1Δ*/Cbk1_T649E	This study

S2 Table. ***Colletotrichum orbiculare*** **strains used in this study.**

Strain	Genotype description	Reference
WT/Cbk1-GFP	Wild type/Cbk1-GFP	This study
pag1Δ/Cbk1-GFP	Wild type/*pag1Δ*/Cbk1-GFP	This study
hym1Δ/Cbk1-GFP	Wild type/*hym1Δ*/Cbk1-GFP	This study
cac1Δ	Wild type/*cac1Δ*	Yamauchi et al., 2004
cac1Δ/Cbk1-CA_1	Wild type/*cac1Δ*/Cbk1_T649E	This study
cac1Δ/Cbk1-CA_2	Wild type/*cac1Δ*/Cbk1_T649E	This study
cac1Δ/Cbk1-CA_3	Wild type/*cac1Δ*/Cbk1_T649E	This study
cmk1Δ	Wild type/*cmk1Δ*	Takano et al., 2000
cmk1Δ/Cbk1-CA_1	Wild type/*cmk1Δ*/Cbk1_T649E	This study
cmk1Δ/Cbk1-CA_2	Wild type/*cmk1Δ*/Cbk1_T649E	This study
cmk1Δ/Cbk1-CA_3	Wild type/*cmk1Δ*/Cbk1_T649E	This study
mekk1Δ	Wild type/*mekk1Δ*	Sakaguchi et al., 2010
mekk1Δ/Cbk1-CA_1	Wild type/*mekk1Δ*/Cbk1_T649E	This study
mekk1Δ/Cbk1-CA_2	Wild type/*mekk1Δ*/Cbk1_T649E	This study
mekk1Δ/Cbk1-CA_3	Wild type/*mekk1Δ*/Cbk1_T649E	This study
