# Supplementary material for: The morphogenesis-related NDR kinase pathway of Colletotrichum orbiculare is required for translating plant surface signals into infection-related morphogenesis and pathogenesis
Source: PLoS Pathog. 2017 Feb 1;13(2):e1006189. doi: 10.1371/journal.ppat.1006189 (PMC5305266; doi:10.1371/journal.ppat.1006189)
Supplement: S3 Table — (PDF) [file ppat.1006189.s011.pdf]

S3 Table. ***Saccharomyces cerevisiae*** **strains used in this study.**

Strain	Genotype description	Reference
W303-1A	*MATa ura3-1 leu2-3,-112 his3-11,-15 trp1-1 ade2-1 can1-100 ssd1-d*	Nelson et al., 2003
tao3Δ (FLY1004)	*MATa CBK1-13myc::HIS3 tao3Δ::KANMX ura3-1 leu2-3,-112 his3-11,-15 trp1-1 ade2-1 can1-100 ssd1-d*	Nelson et al., 2003
tao3Δ/TAO3	*MATa CBK1-13myc::HIS3 tao3Δ::KANMX ura3-1 leu2-3,-112 his3-11,-15 trp1-1 ade2-1 can1-100 ssd1-d pYES2-ScTAO3*	This study
tao3Δ/PAG1	*MATa CBK1-13myc::HIS3 tao3Δ::KANMX ura3-1 leu2-3,-112 his3-11,-15 trp1-1 ade2-1 can1-100 ssd1-d pYES2-CoPAG1*	This study
Y2H Gold	*MATa, trp1-901, leu2-3, 112, ura3-52, his3-200, gal4Δ, gal80Δ, LYS2 : : $GAL1_{UAS}$-$Gal1_{TATA}$-His3, $GAL2_{UAS}$-$Gal2_{TATA}$-Ade2 URA3 : : $MEL1_{UAS}$-$Mel1_{TATA}$ AUR1-C MEL1*	Clontech
Y187	*MATα, ura3-52, his3-200, ade2-101, trp1-901, leu2-3, 112, gal4Δ, gal80Δ, met-,URA3 : : $GAL1_{UAS}$-$Gal1_{TATA}$-LacZ, MEL1*	Clontech
Y2H Gold/ pGBKT7	*MATa, trp1-901, leu2-3, 112, ura3-52, his3-200, gal4Δ, gal80Δ, LYS2 : : $GAL1_{UAS}$-$Gal1_{TATA}$-His3, $GAL2_{UAS}$-$Gal2_{TATA}$-Ade2 URA3 : : $MEL1_{UAS}$-$Mel1_{TATA}$ AUR1-C MEL1 pGBKT7*	This study
Y2H Gold/ pGBKT7-Pag1	*MATa, trp1-901, leu2-3, 112, ura3-52, his3-200, gal4Δ, gal80Δ, LYS2 : : $GAL1_{UAS}$-$Gal1_{TATA}$-His3, $GAL2_{UAS}$-$Gal2_{TATA}$-Ade2 URA3 : : $MEL1_{UAS}$-$Mel1_{TATA}$ AUR1-C MEL1 pGBKT7-Pag1*	This study
Y2H Gold/ pGBKT7-Hym1	*MATa, trp1-901, leu2-3, 112, ura3-52, his3-200, gal4Δ, gal80Δ, LYS2 : : $GAL1_{UAS}$-$Gal1_{TATA}$-His3, $GAL2_{UAS}$-$Gal2_{TATA}$-Ade2 URA3 : : $MEL1_{UAS}$-$Mel1_{TATA}$ AUR1-C MEL1 pGBKT7-Hym1*	This study
Y2H Gold/ pGBKT7-Kel2	*MATa, trp1-901, leu2-3, 112, ura3-52, his3-200, gal4Δ, gal80Δ, LYS2 : : $GAL1_{UAS}$-$Gal1_{TATA}$-His3, $GAL2_{UAS}$-$Gal2_{TATA}$-Ade2 URA3 : : $MEL1_{UAS}$-$Mel1_{TATA}$ AUR1-C MEL1 pGBKT7-Kel2*	This study
Y2H Gold/ pGBKT7-53	*MATa, trp1-901, leu2-3, 112, ura3-52, his3-200, gal4Δ, gal80Δ, LYS2 : : $GAL1_{UAS}$-$Gal1_{TATA}$-His3, $GAL2_{UAS}$-$Gal2_{TATA}$-Ade2 URA3 : : $MEL1_{UAS}$-$Mel1_{TATA}$ AUR1-C MEL1 pGBKT7-53*	This study
Y2H Gold/ pGBKT7-Lam	*MATa, trp1-901, leu2-3, 112, ura3-52, his3-200, gal4Δ, gal80Δ, LYS2 : : $GAL1_{UAS}$-$Gal1_{TATA}$-His3, $GAL2_{UAS}$-$Gal2_{TATA}$-Ade2 URA3 : : $MEL1_{UAS}$-$Mel1_{TATA}$ AUR1-C MEL1 pGBKT7-Lam*	This study
Y187/pGADT7	*MATα, ura3-52, his3-200, ade2-101, trp1-901, leu2-3, 112, gal4Δ, gal80Δ, met-,URA3 : : $GAL1_{UAS}$-$Gal1_{TATA}$-LacZ, MEL1 pGADT7*	This study
Y187/pGADT7-Cbk1	*MATα, ura3-52, his3-200, ade2-101, trp1-901, leu2-3, 112, gal4Δ, gal80Δ, met-,URA3 : : $GAL1_{UAS}$-$Gal1_{TATA}$-LacZ, MEL1 pGADT7-Cbk1*	This study
Y187/pGADT7-T	*MATα, ura3-52, his3-200, ade2-101, trp1-901, leu2-3, 112, gal4Δ, gal80Δ, met-,URA3 : : $GAL1_{UAS}$-$Gal1_{TATA}$-LacZ, MEL1 pGADT7-T*	This study
