# Supplementary material for: The morphogenesis-related NDR kinase pathway of Colletotrichum orbiculare is required for translating plant surface signals into infection-related morphogenesis and pathogenesis
Source: PLoS Pathog. 2017 Feb 1;13(2):e1006189. doi: 10.1371/journal.ppat.1006189 (PMC5305266; doi:10.1371/journal.ppat.1006189)
Supplement: S4 Table — (PDF) [file ppat.1006189.s012.pdf]

S4 Table. PCR primers used in this study.

Name	Sequence (5' → 3')	Note
CoPAG1orf_F3	ATCGGACTACTAGCATCGTCAAAAAATGCACGCCGTTGGT	For pYES2-CoPAG1-cDNA and pYES2-ScTAO3 plasmids
CoPAG1orf_R2	CAGATCTGCTCGACCTGCGGCTCCGGCTCAAAGGG	
CoPAG1orf_F2	GGTCGAGCAGATCTGCGGCCCCGGAGTCGATCCAT	
CoPAG1orf_R1	GAGTCGTATTACAGCGTTTGTCTTCGTGGCAGCGA	
ScTAO3_F1	ATCGGACTACTAGCATAACCCTTTTTAGTTTTGTT	
ScTAO3_R1	GAGTCGTATTACAGCGCCTGCTTTATATTTATTTG	
pYES2_F1	GCTGTAATACGACTCACTATAGGGAATATTAAGCT	
pYES2_R1	TGCTAGTAGTCCGATCCGGGGTTTTTTCTCCTTGA	
g6364_F1A	TCTAGAATTTTTCAACAGCCCTATCGCACT	For pBI-CoPAG1-S and pBI-CoPAG1-SH plasmids
g6364_R2D	ACGTCGACTAAGATGTCGCCTCAAGGGGAG	
g6364_F2C	ACGTCGACTTAGCGTCGCTGATGGTGTAGT	
g6364_R1B	GCTTATCGTCGATGCCTGTACGCTGTGCCG	
g6364_hphF1D	ACATCTTAGTCGACGTTAACTGATATTGAA	
g6364_hphR1C	GACGCTAAGTCGACGTTAACTGGTTCCCGG	
g6364_pBIF1A	GTTGAAAAATTCTAGAAATACGACTCACTA	
g6364_pBIR1B	GGCATCGACGATAAGCTTGATATCGAATTC	
g7445_F1A	TCTAGAATTTTCAGCGTTCACTGTTGCACTCT	For pBI-CoCBK1-B plasmid
g7445_R1B	GCTTATCGTCCAAGTGGCAAGTCATGAACGCC	
g7445_pBIF1A	CGCTGAAAATTCTAGAAATACGACTCACTATA	
g7445_pBIR1B	CACTTGGACGATAAGCTTGATATCGAATTCCT	
g7445_F1A	TCTAGAATTTTCAGCGTTCACTGTTGCACTCT	For pBI-CoCbk1-asB plasmid
g7445_M352A_R1B	TCCCGGCAAGAACTCCGCCAACATGTAGAG	
g7445_M352A_F1A	CTCTACATGTTGGCGGAGTTCTTGCCGGGA	
g7445_R1B	GCTTATCGTCCAAGTGGCAAGTCATGAACGCC	
g7445_M352A_hphF1A	CGTTACATGTCGACGTTAACTGATATTGAA	For pBI-CoCbk1-asBH plasmid
g7445_M352A_hphR1B	TCGTGAGAGTCGACGTTAACTGGTTCCCGG	
g7445_M352A_pBIF1A	ACGTCGACATGTAACGAACCTCTAGCCGCC	
g7445_M352A_pBIR1B	ACGTCGACTCTCACGAGCAGCATGGTGGAC	
g7445_M352A_surF1A	CGTTACATGTCGACGTGCCAACGCCACAGT	For pBI-CoCbk1-asBS plasmid
g7445_M352A_surR1B	TCGTGAGAGTCGACGTGAGAGCATGCAATT	
g7445_M352A_pBIF1Asur	ACGTCGACATGTAACGAACCTCTAGCCGCC	
g7445_M352A_pBIR1Bsur	ACGTCGACTCTCACGAGCAGCATGGTGGAC	

S4 Table. PCR primers used in this study.

Name	Sequence (5' → 3')	Note
g7445_F1A	TCTAGAATTTTCAGCGTTCACTGTTGCACTCT	For pBI-CoCbk1-caBS plasmid
g7445_T649E_R1B	GTCGAAACGCTTGAACTCGTAGCCGATGAAGGG	
g7445_T649E_F1A	CCCTTCATCGGCTACGAGTTCAAGCGTTTCGAC	
g7445_R1B	GCTTATCGTCCAAGTGGCAAGTCATGAACGCC	
g7445_pBIcompF1	CGAGCTGTACAAGTAATGATGTGCTGGGGCCGCGCTGGTG	For pBI-CoCbk1-GFP-B plasmid
g7445_pBIcompR1	TTCCTCCTCCTCCTCCACGGAAGTTGTTGTCGAAACGCTT	
glyGFPF1	GGAGGAGGAGGAGGAATGGTGAGCAAGGGC	
GFPR1	TTACTTGTACAGCTCGTCCATGCCGAGAGT	
g4172_F1A	TCCCTTAATTCTCCGGCTTCAGTACAATCCACGCT	For pBI-CoHYM1-S and pBI-CoHYM1-SH plasmids
g4172_R2D	GCTCCTTCAATATCAGCCGACAAAGCGGAAGCGGC	
g4172_F2C	GCCGACCGGGAACCAGCAGAGCTCGAGCAGCACTT	
g4172_R1B	CAATCTGATCATGAGTATCCAAGCAACTACCGCGA	
g4172_hphF1D	TGATATTGAAGGAGCATTTTTTGGGCTTGGCTGGA	
g4172_hphR1C	TGGTTCCCGGTCGGCAGCGAAAGCGAGAGGGTTGG	
g4172_pBIF1A	CTCATGATCAGATTGTCGTTTCCCGCCTTCAGTTT	
g4172_pBIR1B	CGGAGAATTAAGGGAGTCACGTTATGACCTCTAGT	
Y_PAG1_F1	CATGGAGGCCGAATTCATGCACGCCGTTGGTCGAC	For pGBKT7- and pGADT7- plasmids
Y_PAG1_R1	GCAGGTCGACGGATCCCTACAGCATCTCCCCTTGA	
Y_HYM1_F1	CATGGAGGCCGAATTCATGAAATTCATTCTCCAAG	
Y_HYM1_R1	GCAGGTCGACGGATCCCTAGCTACCCCCAGGAGTG	
Y_KEL2_F1	CATGGAGGCCGAATTCATGGCTTTTCTCTTCAAGT	
Y_KEL2_F1	GCAGGTCGACGGATCCCTAAGGCCGATCGGGACGG	
Y_CBK1_AD_F1	GGAGGCCAGTGAATTCATGGATAACAATAATAACC	
Y_CBK1_AD_R1	TCATCTGCAGCTCGAGTCAACGGAAGTTGTTGTCG	
pGB_F1	GGATCCGTCGACCTGCAGCGGCCGCATAACTAGCA	
pGB_R1	GAATTCGGCCTCCATGGCCATATGCAGGTCCTCCT	
pGD_F1	CTCGAGCTGCAGATGAATCGTAGATACTGAAAAAC	
pGD_R1	GAATTCACTGGCCTCCATGGCCATATGAGCGTAAT	
